# The concept for innovative Comprehensive Assessment of Lowland Rivers

**DOI:** 10.1371/journal.pone.0282720

**Published:** 2023-03-09

**Authors:** Joanna Kocięcka, Jerzy Mirosław Kupiec, Mateusz Hämmerling, Daniel Liberacki

**Affiliations:** 1 Department of Land Improvement, Environmental Development and Spatial Management, Poznań University of Life Sciences, Poznań, Poland; 2 Department of Ecology and Environmental Protection, Poznań University of Life Sciences, Poznań, Poland; 3 Department of Hydraulic and Sanitary Engineering, Poznań University of Life Sciences, Poznań, Poland; KFUPM: King Fahd University of Petroleum & Minerals, SAUDI ARABIA

## Abstract

Current river assessment methods focus on evaluating a single aspect (e.g. the physical and chemical quality of the water or its hydromorphological state) and usually do not integrate various factors. The lack of an interdisciplinary method makes it difficult to correctly assess the condition of a river as a complex ecosystem significantly influenced by humans. This study aimed to develop a novel Comprehensive Assessment of Lowland Rivers (CALR) method. It is designed to integrate and evaluate all-natural and anthropopressure-related elements that influence a river. The CALR method was developed using the Analytic Hierarchy Process (AHP). The application of the AHP allowed the assessment factors to be determined and given weights to define the importance of each assessment element. As a result of AHP analyses, the following ranks were determined for the six main parts of the CALR method: hydrodynamic assessment (0.212), hydromorphological assessment (0.194), macrophyte assessment (0.192), water quality assessment (0.171), hydrological assessment (0.152) hydrotechnical structures assessment (0.081). In the comprehensive assessment of lowland rivers, each of the six elements listed above is rated on a scale of 1–5 (where 5 means very good and 1 bad) and multiplied by an appropriate weighting. After summing up the obtained results, a final value is obtained, classifying the river. CALR can be successfully applied to all lowland rivers thanks to its relatively simple methodology. The widespread use of the CALR method may facilitate the assessment process and enable the comparison of the condition of lowland rivers worldwide. The research conducted in this article is one of the first attempts to develop a comprehensive method for evaluating rivers that considers all aspects.

## 1. Introduction

In times of progressive degradation of the environment, rapidly used natural resources and more and more perceptible climate changes, rational management of water resources becomes the need of the first order. Available water is decreasing at an alarming rate due to its increased use for economic and domestic purposes. Increased transpiration causes water loss from reservoirs, and higher temperature accelerates the processes of surface water degradation [1]. A comprehensive view of the aquatic ecosystem using integral tools is needed to effectively counteract the degradation of aquatic ecosystems, assess their condition, or plan their use or rehabilitation. There are many methods in the world to assess different aspects of rivers. However, they focus mainly on assessing its ecological status, which consists of biological elements, physico-chemical indicators, and hydromorphological conditions. The above parameters have been included and defined in the European Water Framework Directive [2]. Currently, this document is the main legal act regulating the regulations related to the sustainable management of water resources in catchments. It is also the basis for planning, using, and protecting water resources in the European Union (EU).

Over the last 50 years, water demand in Europe has grown. This led to an overall reduction of 24% of renewable water resources per capita in the EU. This decline is particularly evident in southern Europe. The European Environment Agency (EEA) [3] estimates that around one-third of the EU territory is exposed to permanent or temporary water deficiency. This applies to both southern countries (Greece, Portugal, and Spain), which are already experiencing drought problems in the summer, and northern countries, including the United Kingdom, Germany, and Poland.

Fresh water is used not only for the living needs of people but also in agriculture, forestry, industry, energy, tourism, and other service sectors. World population growth, urbanization, environmental pollution, and the effects of climate change place enormous pressure on water resources [4]. Water resources depend on natural conditions, so we cannot influence their amount. About 80% of freshwater consumed in Europe comes from rivers and groundwater, making these sources vulnerable to overexploitation, pollution, and climate change risks. According to the EEA’s water consumption index, on average, around 243,000 hm^3^ of water is spent on economic activity in Europe per year. Although over 140,000 hm^3^ is returned to the environment, it is often polluted [3]. Agriculture uses around 40% of the total water consumed annually in Europe and will continue to be the largest consumer in the years to come. Energy production entails consumption of approx. 28%. In addition, it is also used to generate hydropower. Mining and industry account for 18% of water consumption, of which the textile industry is one of the most water-consuming. Households account for around 12% of water consumption. On average, European households are supplied with 144 litres of water per person per day [5, 6].

Increasingly, the state of water quality and its availability depend on extreme phenomena that have intensified in recent years due to progressive climate change. Water should be available to all organisms. It is an essential element of ecosystem processes and should be used sustainably [7]. Water scarcity is one of the biggest problems today. It should be mentioned that only 2.5% of the total water in the world is freshwater. Of this amount, only 0.3% of all freshwater on Earth is available for use as surface water. The research conducted by the United Nations in Resolution A/RES/71/222 [8] shows that the world may experience a 40% decrease in water availability by 2030 if current trends in water consumption and water resource management continue at current levels. In addition, the United Nations notes that over 2 billion people currently live in conditions of limited access to freshwater resources [9, 10]. In 2050, according to forecasts, at least one in four people in the world will be affected by insufficient freshwater. The forecasts for cereal production worldwide are also worrying, where about half of all production will be at risk of water deficit. The growing human population, increasing climate variability, and improper management of water resources and pollution cause these factors to contribute to the increased water deficit [11]. The increase in catastrophic events such as floods and droughts is essential for the sustainable management of water resources. Increasingly occurring weather anomalies, causing extreme climatic events, increase the risk of flooding [12, 13] and drought [14]. Floods and droughts cause high social and economic costs in various world regions [7].

The changing demographic and economic reality, climate, and environmental problems generated by unsustainable human activity necessitate a detailed inventory and valorization of river ecosystems with a more detailed approach as the basis for planning the protection and shaping of water resources. EU policy aims to significantly reduce pressure on water resources and ensure a sufficient quantity of good-quality water for human and environmental use. River valleys being places of water flow and accumulation, apart from their production function, play an important role in the water management of adjacent areas. This applies to both natural and semi-natural ecosystems as well as agro-ecosystems. Wet and wetland ecosystems are susceptible to climate change and water fluctuations [15]. Here, even a periodic water shortage can cause irreversible changes in the species composition of plant and animal organisms [16]. A disrupted small water cycle, which depends on the availability and quality of surface waters, may also limit agricultural development and diminish ecosystem services [17]. To quantify certain processes related to the production of various products, indicators, for example, virtual water, also called "embedded water" or "indirect water," are introduced, i.e. the amount of water needed to produce a specific product or provide a service. The concept was presented in 1993 by John Anthony Allan of King’s College London and the School of Oriental and African Studies. The water footprint has three components: green, blue, and grey. The first two concern water consumption. A green water footprint is the volume of rainwater used to produce a given product—this applies in particular to agricultural and forestry crops. The blue water footprint is the volume of used surface water and groundwater that has become part of the product in question and the volume of water that has evaporated into the atmosphere through the product’s manufacture. The last component of the water footprint relates to water pollution [18].

Water resources also determine the development of agriculture. In addition, they affect plant production (yield, quality of the grass sward) and animal production (providing water for the livestock’s living needs). As a result, it is necessary to immediately take action to solve the so-called "red light" problems related to climate change, reduction of biodiversity, water deficiency, and the negative impact of pollutants on humans, water and water-dependent ecosystems. Recognition of rivers’ environmental potential and natural, hydrological, hydromorphological or hydrotechnical features will enable the proper design and implementation of a water protection and reclamation plan. Moreover, it will enable the application of practices contributing to maintaining the good condition of rivers, which are the primary water source for ecosystems and humans.

Water is currently becoming a scarce good and, in many cases, a problematic product, resulting from the increasingly frequent extreme phenomena (floods, violent precipitation, droughts, and eutrophication). Undertaking protective and reclamation measures and restoration measures requires a very detailed inventory and valorization of reservoirs, which in their approach will take into account the largest possible number of biotic and abiotic elements and allow for a comprehensive approach to the assessment of their condition. However, some elements are correlated with each other, such as the presence of macroinvertebrate, fish or macrophytes, with chemistry or hydromorphology [19]. Thus, troublesome analyzes of some biotic parameters may be omitted because the large hydromorphological diversity, optimal chemical parameters, or the large biodiversity of macrophytes indicate the high biological potential of the reservoir. Therefore, the authors of this study noticed a gap in science concerning the river assessment methods used. The methods currently used focus on one aspect. In most cases, it is water quality or watercourse hydromorphology. Existing methods do not try to integrate many different factors and assess their impact in the context of the functioning of the entire river. It is impossible to compare the status of all lowland rivers using a single method that considers all significant parameters for this ecosystem. Consequently, a tool is lacking to assess and compare rivers holistically. The authors of this article have attempted for the first time to develop a novel, comprehensive method of assessing lowland rivers that consider factors including both natural parameters and human impacts on the river. This research is particularly significant because of the need to use a uniform scale to compare the current state of rivers to develop water management plans for large areas such as the European Union. A standardized methodology for assessing lowland rivers will allow all rivers in the world to be correctly listed and compared. Through its widespread use, governments and the public will become aware of watercourses needing immediate water management intervention. Furthermore, the method will allow for a better scientific understanding and inventory of watercourses. Its application will enable many scientists to comprehensively and accurately study many rivers and draw conclusions about their current state worldwide. Such studies will significantly enhance knowledge of hydrology, hydromorphology, water quality, and many other rivers-related fields.

This study aimed to develop a comprehensive method for assessing lowland rivers. A method that will integrate environmental aspects and the impact of anthropopressure on the river. The main assumptions of the method proposed by the authors are universality and ease of application to all lowland rivers. The river assessment tool developed by the authors of this study is based on calculations made using the Analytic Hierarchy Process, which makes it possible to classify the importance of individual river assessment elements. The novelty of the method also lies in the fact that, for the first time, so many factors are taken into account when assessing rivers, and their importance is hierarchized. The methodology presented in this paper provides a practical guide for assessing lowland rivers worldwide. Furthermore, the implication of the method on a global scale will allow standardising currently used various assessment methods and comparing the state of lowland rivers on a global scale. Therefore, its application will be very helpful in developing plans for appropriate water resource management on a local and global scale. The correct preparation of documents defining water management methods in the coming years is crucial in view of the ongoing climate change and the need to save water in many scarce regions of the world.

## 2. Materials and methods

**Fig 1:**
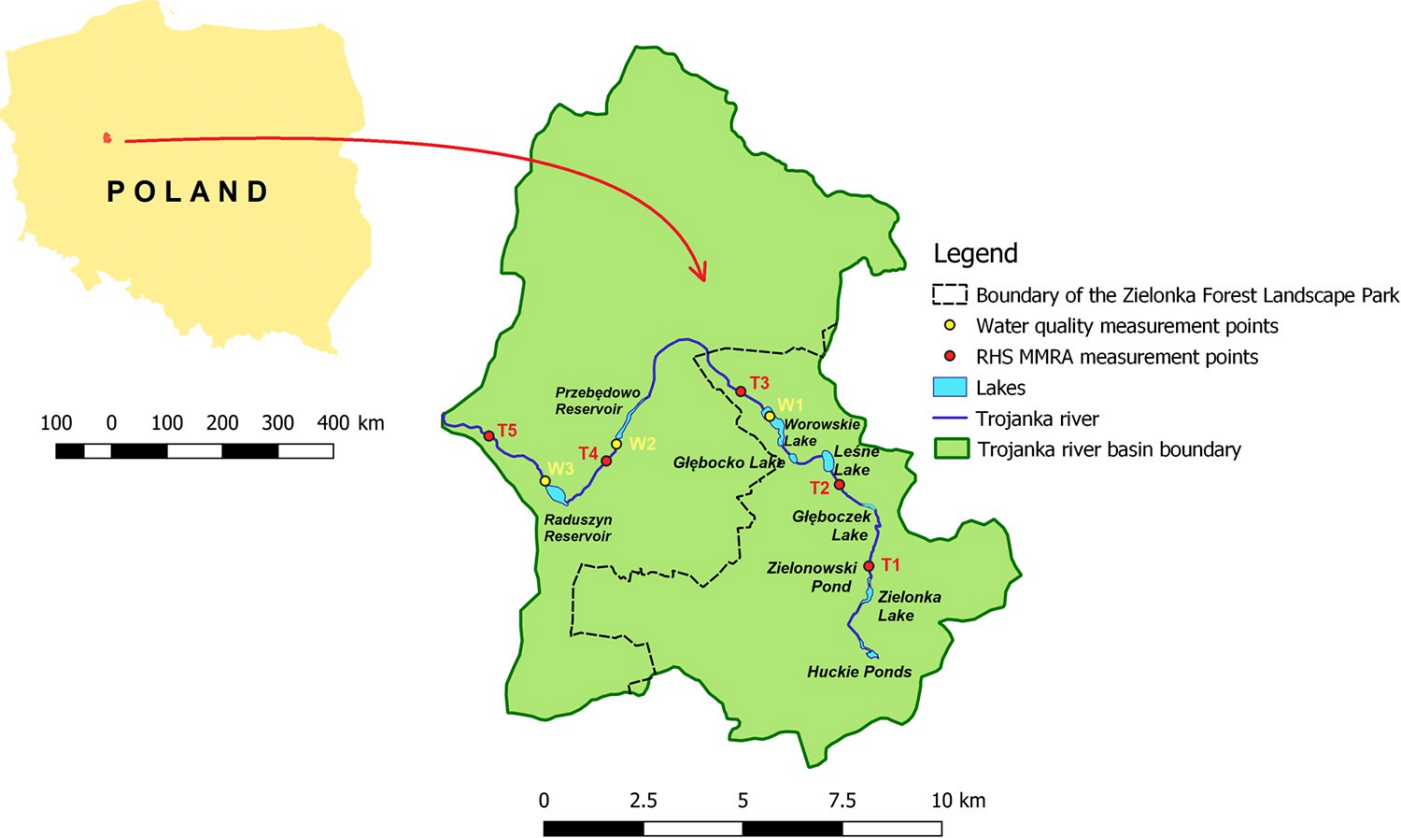
Trojanka River catchment area (shapefile source: https://dane.gov.pl/).

**Fig 2:**
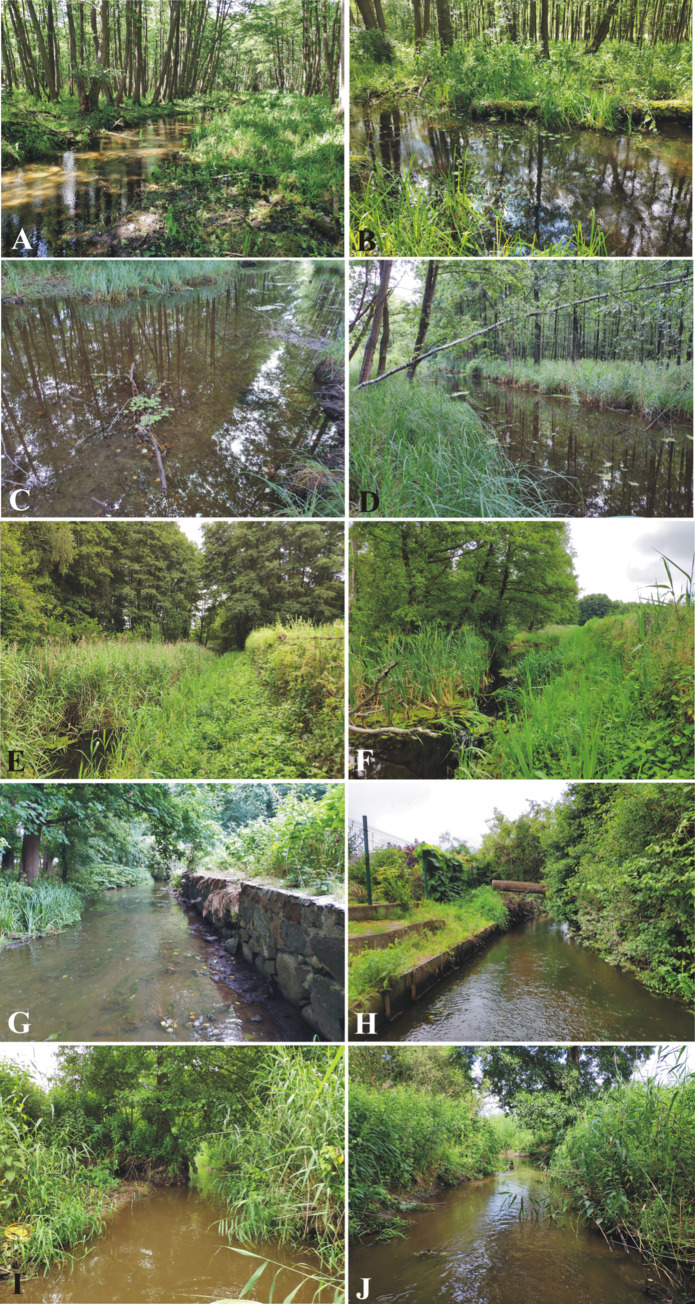
Analyzed research cross-sections on the Trojanka river: A&B—point T1; C&D—point T2; E&F—point T3; G&H—point T4; I&J—point T5.

Several field measurements were previously carried out to develop a comprehensive method for assessing lowland rivers. As a research object, the Trojanka River was chosen. The river can be found in Poland within the Oder river basin, the Warta Water Region. The total length of the river is 20.8 km. Its spring is located in the area of Huckie Ponds in the protected area of the Zielonka Forest Landscape Park, and its mouth is the Warta River. The Trojanka River flows through the following lakes: Zielonka Lake, Zielonowski Pond, Głęboczek Lake, Leśne Lake, Głębocko Lake, Worowskie Lake, Przebędowo Reservoir and Raduszyn Reservoir (Fig 1). The Trojanka catchment has a lowland character and is situated in the central part of Poland, included in the Central European Lowlands. The river is described by a separate EU Surface Water Body Code PLRW600017185969. Like most rivers in this region, it has ground, rainfall, and snowfall recharge. The gradient of the river is 1.28‰. The total catchment area of the Trojanka is 145.34 km^2^. The average unit outflow from the catchment estimated by scientists ranges from 2.5 dm^3^·s^-1^·km^-2^ to 3.73 dm^3^·s^-1^·km^-2^ [20, 21]. Based on the abiotic typology of flowing waters, Trojanka is classified as a lowland sandy brook (Type 17). According to the Water Framework Directive, the term stream is used for small rivers (100–1000 km^2^). However, it should be noted that a significant majority, as many as 1788 Individual Bodies of River Surface Waters in Poland, are classified exactly as this Type 17 [22]. Consequently, this river was selected by the authors of this publication as the representative to demonstrate a Comprehensive Assessment of Lowland Rivers (CALR) on its example. The visual characteristics of the test cross-sections (T1-T5) of the Trojanka River are presented in Fig 2.

**Fig 3:**
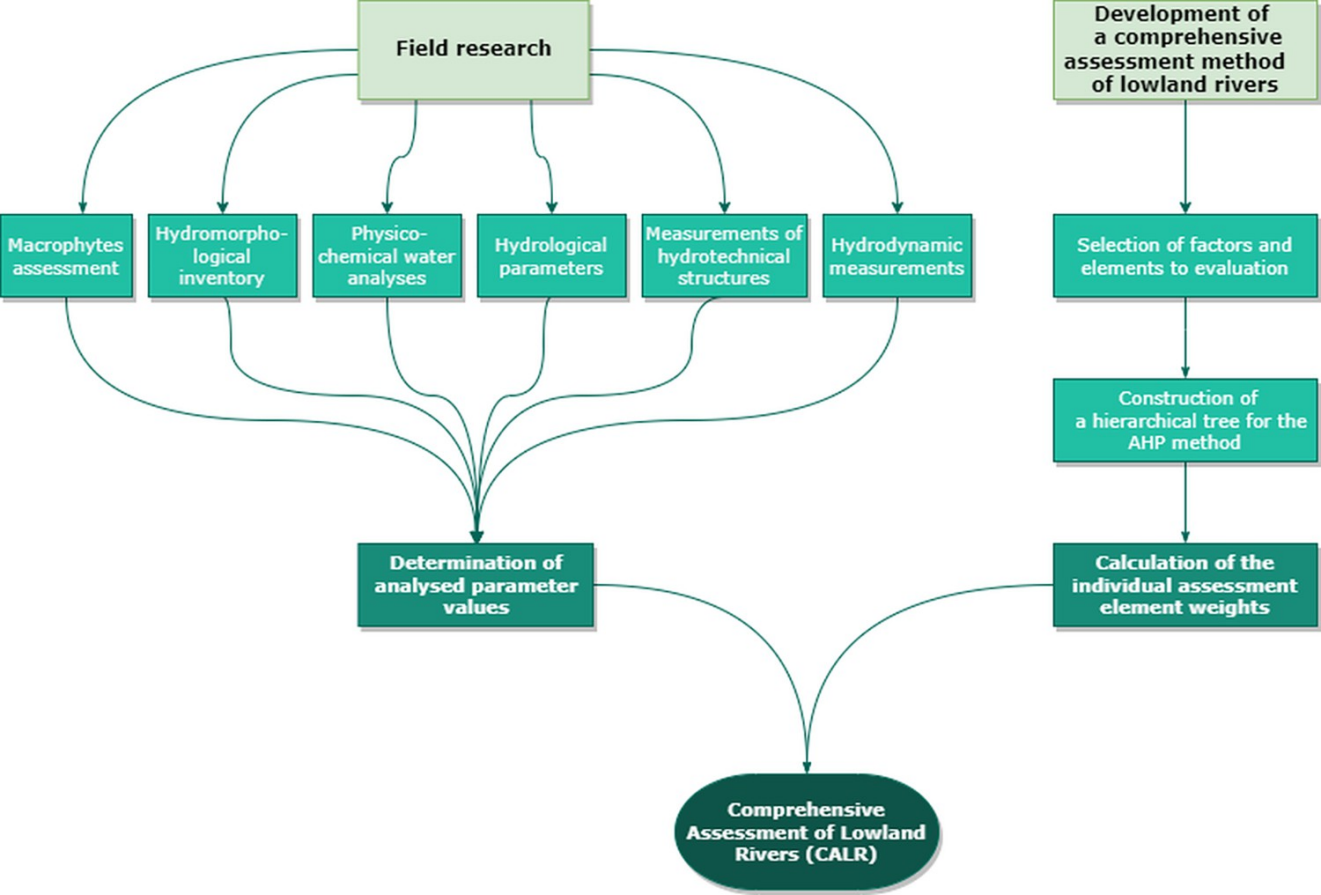
Workflow chart.

As part of the field studies conducted on the Trojanka River, several parameters were measured and evaluated for future use in the Comprehensive Assessment of Lowland Rivers (CALR). No permit was required to carry out the field survey. The study area is open to the public, and measurements can occur at any time and location on the watercourse. The field investigations included hydrological and hydrodynamic measurements, the Macrophyte Method for River Assessment (MMRA), River Habitat Survey (RHS), and the evaluation of the technical condition of hydrotechnical structures. Water quality was also assessed using data from the Provincial Environmental Protection Inspectorate in Poznań. Furthermore, to develop a Comprehensive Assessment Method for Lowland Rivers, the factors critical for this assessment were selected in cooperation with experts in the following fields: hydrology, river engineering, hydrotechnical engineering, land reclamation, and river ecology. The Analytic Hierarchy Process (AHP), a multi-criteria method for analysing decision-making problems, was used for this purpose. The detailed scheme of the authors’ research and analysis in this manuscript is presented in the flow chart (Fig 3). The assessment of each of the above-mentioned important aspects of the river (RHS, MMRA, hydrology, hydrodynamic, water quality and condition of hydrotechnical structures) was based on a five-point scale, which is included in S1 Table.

### 2.1. Field research

#### 2.1.1. Macrophytes assessment

Bioindication studies in selected control sections were performed using the Macrophyte Method for River Assessment (MMRA) [23]. The method is based on the qualitative and quantitative assessment of the species composition of macrophytes present in the water. It allows for determining the degree of degradation of the watercourse, mainly in relation to its trophic state. The research was carried out on 100-meter representative sections of the analyzed river. MMRA was assessed at five points: T1 named, Zielonka” (52°33’26.05"N, 17°06’40.76"E), T2:, Głęboczek” (52°3428.82" N, 17°06’06.92"E), T3:, Gębice” (52°36’12.46"N, 17°02’58.09"E), T4:, behind the Przebędowo reservoir” (52°35’06.55"N, 17°00’55.05"E), T5:, Mściszewo” (52°35’05.54"N, 16°58’14.95"E). The locations of the points are shown on the map in Fig 1. In the MMRA study, only macrophytes growing in water (at least rooted in water or in the so-called splash zone) were recorded. According to the methodology, macrophytes were identified to the species level or genus, in the case of algae. 189 indicator taxa are used to calculate the MRI index, including 63 monocotyledonous vascular plants, 55 dicotyledons, 5 ferns, 27 mosses, 13 liverworts, 1 water lichen (*Collema*), 22 macroalgae, 2 cyanobacteria and 1 colony bacterium (*Sphaerotilus natans*). For each taxa, the share in watercourse coverage was recorded using the following nine-point scale: 1 for <0.1%, 2 for 0.1–1%, 3 for 1–2.5%, 4 for 2.5–5%, 5 for 5–10%, 6 for 10–25%, 7 for 25–50%, 8 for 50–75% and 9 for >75%. Based on the inventory, the Macrophyte River Index (MRI) was calculated, which, when related to the reference values for a given macrophyte type, allows for the assessment of the ecological status defined by the Water Framework Directive [2, 24].

The total number of species was assessed by direct identification in the field. Species that grew in the water or amphibious species growing on the watercourse bank, leaning towards the water, are included.

Indicator species were identified based on the list of macrophyte species used in the MMRA. The basic criterion for selecting species for the index list is the trophic level of the aquatic environment in which a given taxon occurs (from advanced trophic to oligotrophy). Additionally, the criterion is the ecological tolerance of the species (from sten- to eurotypic). Indicator plants and all other aquatic plants were identified in a 100-meter stretch of the watercourse.In addition, the degree of coverage was assessed based on all species of aquatic plants present in the riverbed.

#### 2.1.2. Hydromorphological inventory

**Fig 4:**
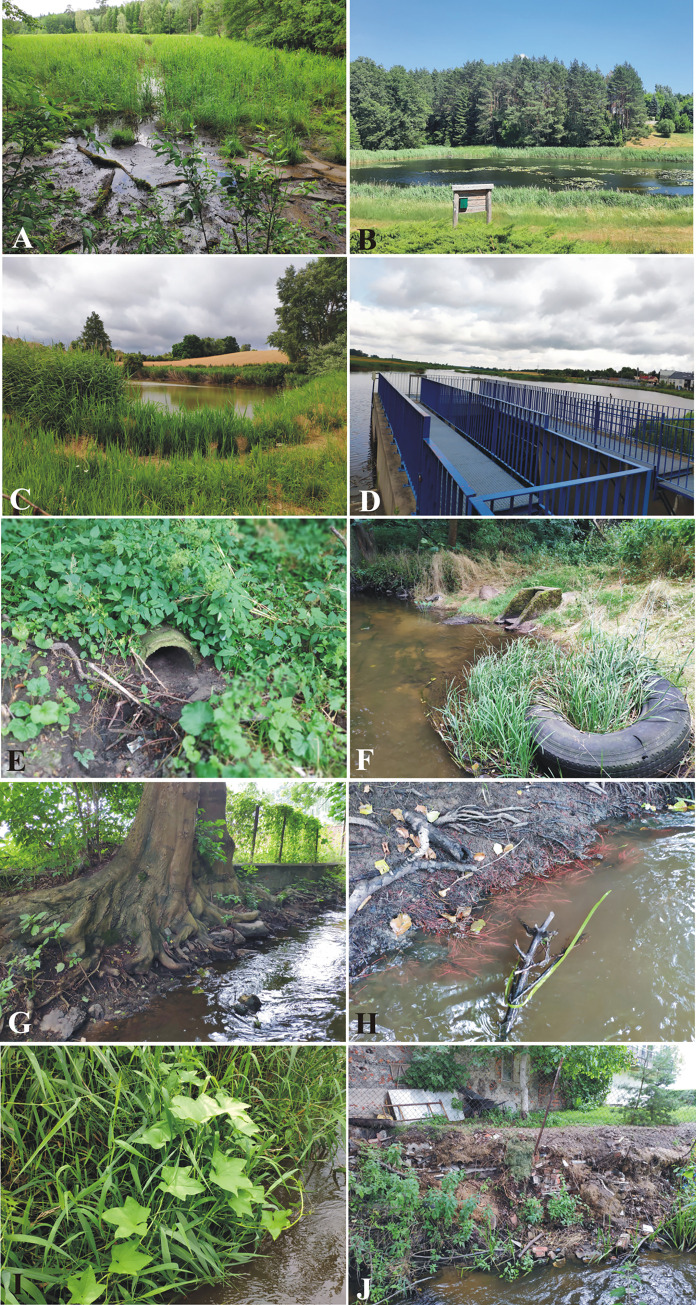
Selected hydromorphological features of the river bed and its immediate surroundings observed during the research: A—Trojanka river water-head area; B—Zielonowski Pond; C—a fish pond near point T3; D—“Przebędowo” dam reservoir; E—sewage discharges in the vicinity of point T4; F—waste and rubbish in the vicinity of T4; G—old trees and bare roots on the river bank at point T4; H—underwater tree roots near point T5; I—alien species attesting to the anthropization of the habitat (point T5); J—the bank of the river made of waste near point T5.

Hydromorphological studies were carried out using the River Habitat Survey (RHS) method. The assessment of the hydromorphological status of rivers is one of the tasks resulting from the implementation of the Water Framework Directive procedures throughout the European Union. It is a tool for determining the reference conditions of the river and planning activities in the management of the catchment area, including flood protection and river restoration. The RHS inventory was performed on selected 500-meter representative sections of the analysed river [25]. The same locations were used for measurements as in the MMRA (Fig 1). The research was carried out in two stages following the methodology. In the first stage, the morphological features of the riverbed and banks were characterised in 10 control profiles spaced every 50 m. The scope of the inventory also includes the water and bank vegetation structure and the nature and use of the river valley. In the second stage, a synthetic characterization of the entire research section was made, in which morphological forms and transformations not previously recorded in 10 profiles were taken into account. The RHS method allows for a very detailed hydromorphological characterization of the watercourse. It allows the identification of about 400 morphological parameters of the river. Two synthetic indicators (Habitat Quality Assessment—HQA, Habitat Modification Score—HMS) were calculated based on the collected field data. The naturalness index (HQA) allows for assessing the diversity of natural elements of the riverbed and valley. The assessment components are the physical parameters of the riverbed, bank features, types of vegetation in the riverbed, vegetation structure on slopes, trees and land use at a distance of 50 m from the shore. The habitat transformation index (HMS) was calculated based on the type and number of water structures, bank modifications, channel profile transformations, and disturbances in the valley’s water relations. Examples of hydromorphological parameters and catchment features observed during the research are shown in Fig 4.

#### 2.1.3. Physico-chemical water analysis

In addition to the RHS and MMRA assessments, the water quality of the Trojanka River was also assessed. The data came from the state monitoring conducted by Regional Environmental Protection Inspectorate (REPI) in Poznań. Sampling locations for water quality analysis are marked in Fig 1. The analyses used microbiological indicators such as Coliform bacteria, Escherichia Coli and Enterococci. For the indicators mentioned above, a valuation scale based on the division into classes was used (Table 1). For enterococci and E. coli, the Regulation of the Minister of Health of 8 April 2011 on the supervision of water quality in the bathing area and in the place used for bathing was used [26]. For coliform bacteria, the Regulation of the Minister of the Environment of February 11, 2004 on the classification for presenting the status of surface and ground waters, the method of monitoring and the method of interpreting the results and presenting the status of these waters was used [27], due to the lack of this parameter in the later regulation.

**Table 1:** Bonitation for selected microbiological and physico-chemical parameters.

Microbiological parameters					
Specification	NPL·100 ml^-1^				
Class	I	II	III	IV	V
Coliform bacteria	50	500	5000	50000	>50000
Bonitation	5	4	3	2	1
Escherichia coli	200	400*	330**	-	-
Enterococci	500	1000*	900**	-	-
Bonitation	3	2	1	-	-
Physical and chemical parameters					
Specification	mg·L^-1^				
Class	I	II	III	IV	V
Dissolved oxygen (DO)	≥7	6	5	4	<4
Total nitrogen	2.5	5	10	20	>20
Total phosphorus	0.2	0.4	0.7	1	>1
Bonitation	1	2	3	4	5

* based on a 95^th^ percentile assessment

* based on a 90^th^ percentile assessment

The analyses included nutrients such as total nitrogen and total phosphorus in the water. The score evaluation was based on the Regulation of the Minister of the Environment of February 11, 2004, on the classification for presenting the status of surface and ground waters, the method of monitoring and the method of interpreting the results and presenting the status of these waters [27]. The current Regulation of the Minister of Maritime Economy and Inland Navigation of October 11, 2019, on the classification of ecological status, ecological potential and chemical status and the method of classification of the state of surface water bodies, as well as environmental quality standards for priority substances [28], covers only very good and good classification. The other classes do not stand out. Therefore, an older, 5-step classification was adopted for the analyses, which facilitated the performance of the scoring evaluation (Table 1). Dissolved oxygen was also analysed according to the Regulation of the Minister of the Environment of February 11, 2004 [27], which included a 5-level classification of surface waters.

The multiplicity of exceeding the norm for microbiological and physico-chemical parameters was calculated based on the reference annual average concentrations in relation to class II (good condition—in accordance with the EU Water Framework Directive).

#### 2.1.4. Hydrological parameters

Another analysis conducted on the Trojanka River was a hydrological assessment. It was performed based on the modified hydric significance (HS) index [29]. It takes into account parameters such as:

- Meteorological conditions–the amount of precipitation (PP)
- Geomorphology–slope (S)
- Soil conditions–soil types according to wetness index (Swet), soil texture (Stext)
- Hydrogeology–ground transmissivity (T)
- Land-cover characteristics–current land-use (LU)
- The forest cover characteristics–forest ecological status (F)

In the modified version of the HS index for this publication, the slope parameter was removed, and the lake retention (LR) was added in its place. The slope factor in the original method version is referred to as the catchment slope. However, in this method, only the lowland rivers are assessed as watercourses (without catchment areas); hence the values of this parameter would be similar in all cases, which is why this parameter was dropped. Instead, the lake retention index was added to reflect more precisely the actual retention status of the river, which is the main element of the hydrological assessment. Lake retention was calculated based on field bathymetric measurements. For reservoir retention, a scale was used [30]:

- micro reservoirs: capacity < 100 000 m^3^, damming height < 1 m;
- small reservoirs: capacity < 500 000 m^3^, damming height 1m < 5 m;
- medium reservoirs: capacity < 1 million m^3^, damming height 5m < 15 m;
- large reservoirs: capacity > 1 mln m^3^, damming height > 15 m.

The following scores have been given:

- micro reservoirs 0.5 points;
- small reservoirs 1.0 points;
- medium reservoirs 1.5 points;
- large reservoirs 2.0 points.

Other parameters were assessed according to the HS methodology proposed by Šatalová et al. [29] based on maps and satellite data in the QGIS environment. Satellite images, forest damage level maps, soil maps, and topographic maps were used for this purpose. The authors’ main goal of this publication was to assess the condition of the river, so the calculation of the HS index was related to the river length and not to the catchment area as in the original methodology.

The final HS indicator value was calculated based on the formula:

HS=3.5LR+1.5T+2.5Swet+3Stext+4PP+2LU+1F (1)

The scale proposed by Šatalová et al. [29] with modification was used to assess HS. The modification included an adjustment to a 5-degree scale, which is necessary for a comprehensive assessment of lowland rivers:

HS ≥ 20.5, excellent, grade 5.00;

HS = 15.5–20.0, very good, grade 4.00;

HS = 10.5–15.0, good, grade 3.00;

HS = 0.5–10.0, average, grade 2.00;

HS ≤ 0, limited, grade 1.00

#### 2.1.5. Measurements of hydrotechnical structures

In the case of the Trojanka River, the technical condition of hydraulic structures was assessed using the Kaca and Interewicz method [31, 32]. This method assesses individual structure elements such as abutments and their backfill, lifting mechanism, sluice, sluice guide, impervious apron, downstream and upstream apron, building signposting, anti-corrosion and start-up protection, footbridge on the valve, and sealing. The measured parameters are evaluated using tables specifying the limit values for each parameter. Then, every element is classified. The classification is based on a modified scale with the following technical conditions: good condition = 5, satisfactory condition = 3, and unsatisfactory condition = 1. The final grade for each object is calculated as the average of the scores obtained for each element. Research has shown that the Kaca and Interewicz method is effective and suitable for assessing small structures but should not be used for large hydraulic structures such as dams [32]. The authors decided to use this method because there are only small structures, such as weirs, small sills, and culverts, on the Trojanka River. The comprehensive method of assessing lowland rivers presented in this paper does not impose a specific method for evaluating the technical condition of structures. Depending on the nature of the structures (their size, importance in the context of water management and other parameters), a method should be used to reasonably reflect the actual condition of the structure. The Comprehensive Assessment of Lowland Rivers method only assumes that the final condition of all hydrotechnical structures on the river should be determined by a number from a scale of 1–5, where 1 means poor technical condition and 5 means very good technical condition.

#### 2.1.6. Hydrodynamic measurements

The methods presented above cover many areas. In order to increase the accuracy of river assessment, hydrodynamic parameters are also included. There are many relationships under this term. In the analyses, several were selected related to the variability of the riverbed planview, the hydraulics of open channels, the characteristics of the flow volume, and its utility in the context of the life of aquatic organisms in the river. Their classification was made during the hydrodynamic assessment of the river.

A single meander of a naturally flowing river is characterised by a curvature that varies along the length of the arc. The description of the changes in radii and curvature is complex. At the beginning and the end of the curve, the radii of curvature are the largest and the curvature the smallest. The determination of the curvature of the channel is complicated and labour-intensive. Bognar et al.[33] proposed a method for assessing the curvature of rivers based on a comparison of chord length H and curve length L. The types of curvature with assigned rating values can be summarised as follows:

- undeveloped (pseudo-curvature) L<1.1H, assessment 1;
- developed 1.1H<L<1.4H, assessment 2;
- fully developed L>1.4H, assessment 4;
- overdeveloped L>3.5H, assessment 5.

Vegetation is an essential element of a riverbed, influencing water flow dynamics in rivers and channels. It causes a reduction in the active area of the cross-section and a reduction in the velocity of water flow, leading to a reduction in the channel’s capacity. The flow rate also depends on the shape and geometry of the bed and banks of the channel [34]. One of the parameters that help to determine the influence of variability in channel characteristics is the roughness coefficient. Using Ven Te Chow [35], a subdivision of small lowland watercourses was prepared depending on the vegetation present and the channel characteristics for which the width at flood time is no more than 30 m. The ratings depending on the characteristics of the riverbed are summarised below:

- clean, straight, no rifts or deep pools n = 0.030, assessment 1;
- clean, straight, no rifts or deep pools, but more stones and weeds n = 0.035, assessment 2;
- clean, winding, some pools and weeds n = 0.040, assessment 3;
- clean, winding, some pools and weeds, some weeds and stones n = 0.045, assessment 4;
- clean, winding, some pools and weeds, but more stones n = 0.050, assessment 5.

Based on the field analysis of the riverbed, grades were assigned for the different sections of the Trojanka River. The environmental flow was determined based on the ecosystem’s habitat requirements of representative (indicator) species/taxa. The multi-year average flow was calculated separately for October-March and April-September. The measured flow was then checked for the range in Table 2. Based on comparing the tabulated recommended flows with observed flows, ratings for the river were assigned. Table 2 summarises the recommended environmental flows concerning the multi-year average flow [34].

**Table 2:** Summary of river ratings concerning environmental flow.

Water and habitat conditions for flow	Recommended flow rate (% multi-year average flow) m^3^·s^-1^	Recommended flow rate (% multi-year average flow) m^3^·s^-1^	Assessment
	October—March	April—September	
Flood or maximum flow	200%	200%	1
Optimum	60–100%	60–100%	3
Excellent	40%	60%	4
Great	30%	50%	5
Good	20%	40%	3
Acceptable	10%	30%	2
Minimum	10%	10%	1

The final hydrodynamic rating (S1 Table) was determined as the average of the assessments of the individual parameters

Very good hydrodynamic parameters (5.5 > average > 4.5), assessment grade 5.0

Good hydrodynamic parameters (4.5 > average > 3.5), assessment 4.0

Sufficient hydrodynamic parameters (3.5 > average > 2.5), assessment 3.0

Insufficient hydrodynamic parameters (2.5 > average > 1.5), assessment 2.0

Bad hydrodynamic parameters (1.5 > average), assessment 1.0.

### 2.2. Development of a Comprehensive Assessment of Lowland Rivers (CALR)

Choosing the most appropriate method to assess a lowland river is difficult. It requires considering many parameters describing both the influence of hydraulic, environmental, anthropogenic, and other factors. For this type of analysis, it is best to use Multiple-Criteria Decision-Making (MCDM) methods. These methods make it possible to prioritise factors describing a given problem and solutions to the problem. The AHP method, one of the multi-criteria decision support methods, also allows for determining individual solutions’ weights (importance) based on mathematical calculations. The obtained weights and the elements’ ratings will be used to determine one final value of the lowland river assessment. The final score will be obtained by multiplying the partial scores of the components by the weights.

The first step in multi-criteria analyses is to create a hierarchical tree. Next, the factors influencing the analysed problem (level II) should be listed to do this. In the next step, more detailed factors are assigned to level III of the hierarchical tree. Finally, the last level (IV) creates solutions to the problem described at level I of the hierarchical tree.

**Fig 5:**
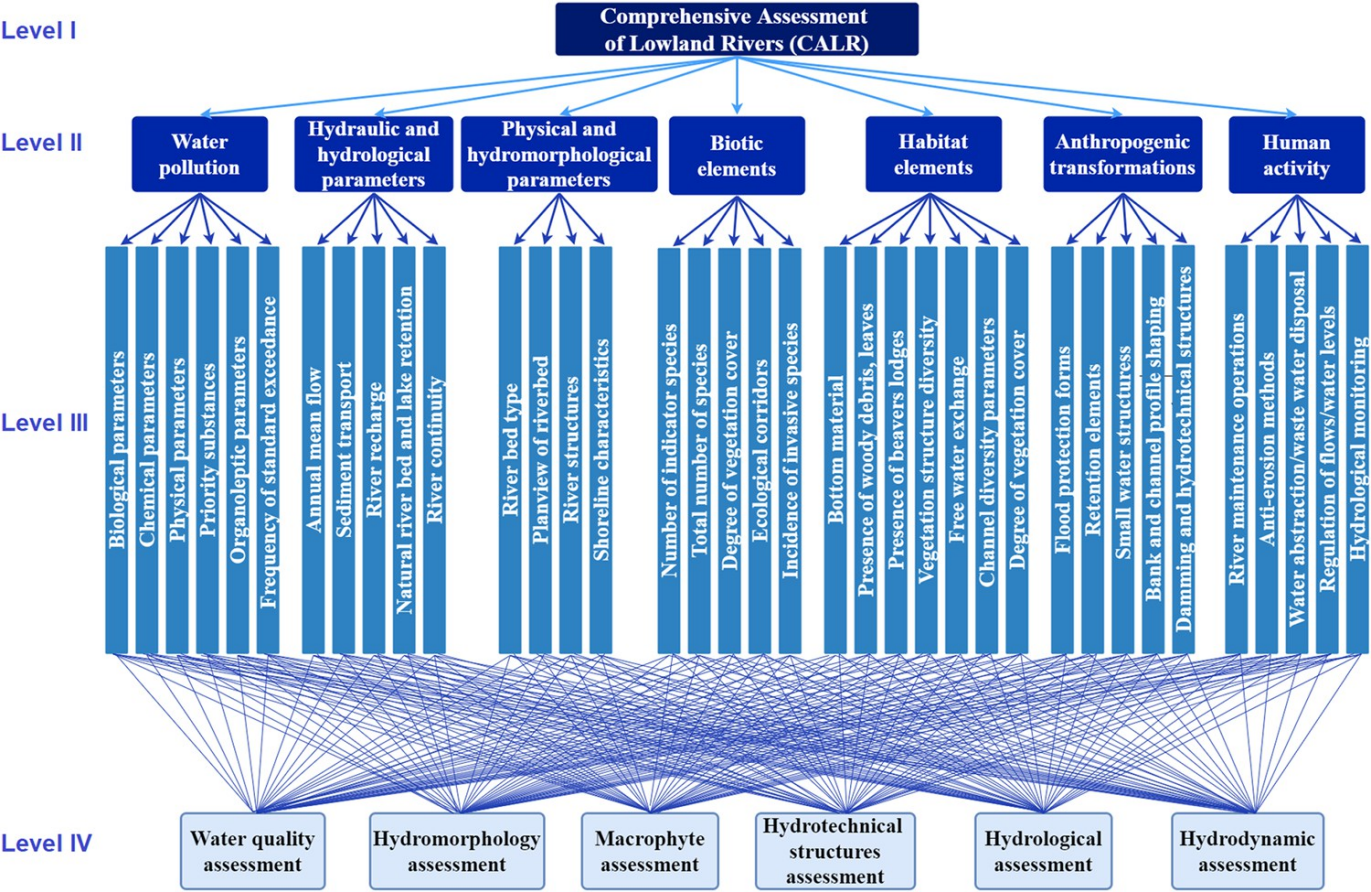
Hierarchical tree for the AHP method.

In creating a hierarchical tree for a Comprehensive Assessment of Lowland Rivers, factors describing the different levels of the tree were selected based on expert knowledge, literature analysis and consultation with other scientists. A wide variety of factors were considered in the presented river assessment method. The authors tried to consider both the natural parameters of the watercourse and the human impact on its transformation. As a result, level II includes the following elements: water pollution, hydraulic and hydrological parameters, physical and hydromorphological parameters, biotic elements, habitat elements, anthropogenic transformations, and human activities (Fig 5).

According to the authors, water pollution is a significant factor affecting a river’s condition. It has negative consequences for humans and aquatic organisms living in the habitat [36]. Therefore, combating the ecological deterioration of rivers is one of the most important challenges. Water quality assessment is influenced by biological, chemical and physical parameters, priority substances, organoleptic parameters, and the multiplicity of exceedance of the standard (scale of exceedance). Physical and chemical environmental factors can affect the biological components of rivers, including benthic macroinvertebrates [37]. One of the factors exerting variations in river water quality is temperature. It is the parameter that influences all river physicochemical changes and biological reactions. It affects dissolution and leads to an increase in the concentration of dissolved substances in the water and a decrease in dissolved oxygen (DO) concentration in the water [38]. River pollution usually occurs gradually and results from natural and anthropogenic changes [39]. The former include climatic changes characterized, for example, by the uneven distribution of precipitation over time and space, which results in changes in stream flows and water temperatures [40]. While the latter include agricultural and industrial effluent emissions and discharge from storm sewers and treatment plants. These cause hydraulic disturbances that affect large river changes, resulting in DO depletion, increased ammonium toxicity, and nutrient and pesticide loading [41]. These changes usually result in a decrease in biodiversity, which is one of the biggest ecological problems threatening aquatic ecosystems [42].

Furthermore, hydraulic and hydrological parameters were considered important in a comprehensive river assessment method. This is because they directly condition the functioning of the watercourse. These parameters distinguished Level III factors: annual mean flow, sediment transport, river recharge, natural river bed and lake retention, and river continuity. The flow rate is the fundamental factor shaping the river channel and enabling living organisms in the river. Flow variability is influenced by season, precipitation characteristics, surface runoff, and catchment properties. Transporting particularly fine sediment is essential regarding ecological feedback on habitat quality and quantity [43]. Too much-transported drift sediment reduces the availability of trophic resources [44], which significantly impacts the organisms living in the rivers. River recharge affects many parameters, such as the amount of available water used for different purposes [45], the intensity of sediment transport, and the ecosystem. Natural retention depends on the slope of the river bed, the vegetation cover, the shape, and the channel regulation state. Even greater changes to the hydraulics and habitat conditions for aquatic organisms are introduced by damming a river [46]. The construction of a barrier causes changes in sediment transport flows and restricts the migration of aquatic organisms [47]. This is the reason why fish ladders are built to allow the movement of aquatic organisms along the watercourse length.

Another element presented at level III of the hierarchical tree is physical and hydromorphological parameters. These include riverbed type, planview of the riverbed, river structures, and shoreline characteristics. The riverbed type is closely linked to the planview of the riverbed. River channel regulation often reduces the river’s length and raises the water table, increasing flood losses. Restoring the natural character of river channels is essential and increases the retention potential of rivers and reduces the frequency and magnitude of flooding during flood events [48]. The shoreline can be shaped by natural or artificial means. For example, bank erosion is reduced when bank reinforcement is used, which results in vertical and horizontal stabilisation of the shoreline [49].

The next group strongly affecting rivers are biotic elements, which determine the functioning of the biological life of the watercourse. As part of their assessment at level III of the hierarchical tree, the following were distinguished: the number of indicator species, the total number of species, the degree of vegetation cover, ecological corridors, and the presence of invasive species. Macrophytes that occur in the aquatic environment are a very sensitive tool for assessing their condition. The presence or absence of indicator plants or plants of wide ecological amplitude provides sufficient information on environmental stress and the ecological condition of water bodies [50]. The occurrence of some species is related to human activities, such as agricultural production. Plant species composition and functional structure often indicate the habitat type and various pressures, including anthropogenic [51]. The aquatic vegetation also builds ecological corridors, which are especially important for animal organisms and the resulting network of biotopes [52]. Invasive species can be found more and more often among aquatic plants. They can reduce the aesthetic and economic value of aquatic ecosystems. They pose a serious threat to water bodies because they are expansive and can displace native species, reduce the effectiveness of ecosystem services and cause significant economic losses. Excessive growth of vegetation, which is invasive, may hinder water movement, affect its physico-chemical state and organoleptic parameters, and in extreme cases, contribute to flooding [53].

Habitat elements were also considered in the comprehensive lowland river assessment method. They affect the biodiversity of the plant and animal world and the creation of microhabitats. Level III factors in this group are bottom material, wood rubble, leaves; lodges; varied structure of vegetation; free water exchange; parameters determining the diversity of channels, and morphological types of flow. The functioning of the aquatic ecosystem is based on the interactions of individual habitat patches and the connectivity between habitats. Some aquatic life stages require different habitats for them to develop properly [54]. Some habitat elements, such as beaver dams or different natural dams, influence the diversification of aquatic habitats and biodiversity [55]. It is similar in the case of free water exchange between individual river sections, the floodplain terrace, and the river. The vegetation structure modifies the occurrence of certain species of animal organisms and affects the dynamics of flows, overflows of the river beyond the riverbed, water speed, sediment transport, and the nature of the riverbed [56]. Morphological types of flow (standing water, scarcely perceptible flow, smooth flow, rippled, unbroken standing waves, broken standing waves, chute) also influence macro-invertebrates’ diversity [57].

Furthermore, the method for assessing lowland rivers also considers human pressure on the watercourse. In this connection, a distinction is made between anthropogenic transformations, including flood defences, retention elements, small aquatic structures, damming/hydrotechnical structures, and the shaping of bank and bed profiles. Moreover, the second group includes human activity, which plays a crucial role in rivers. It can have both positive and negative impacts on the river. For example, human activity has been proven to alter the composition of plankton in river networks [58]. Furthermore, the construction of hydrotechnical structures in the riverbed contributes to morphological changes in the river. For example, it was shown that the channel’s narrowing due to the construction of a ferry crossing caused intensive sedimentation downstream of the narrowing in the expansion zone. In contrast, bank erosion and the channel bar strengthening outwash were observed under other flow conditions [59]. The analyses indicate that hydropower plants can also influence the river’s hydromorphological conditions, although their impact is ambiguous. However, it was observed that in many cases downstream of the power plants, the hydromorphological condition of the river deteriorates [60].

Furthermore, large dams may cause deterioration of the river’s ecological status [58]. Therefore, it is crucial to properly manage hydraulic structures, including dams [61]. In addition, the structures should be maintained in an appropriate technical condition to ensure the functionality of the construction. However, it should be noted that the destruction of bank protections supports natural restoration and forming the braided or anabranching channel pattern [62]. Furthermore, researchers emphasise the importance of integrating flood protection and floodplain restoration [63]. Hence, it can be concluded that assessing human activities on rivers is a very complex problem due to its multifaceted nature. For this reason, the authors of this manuscript have identified as many as five factors in this section.

The last group listed at Level II are human activities that directly affect the river’s transformation. Elements such as river maintenance operations, anti-erosion action, water abstraction/wastewater disposal, and flow/water level regulation are distinguished here. As research indicates, maintaining the natural flow regime in the river affects the condition of aquatic habitats, increases biodiversity and improves the functioning of river ecosystems. Understanding these relationships between channel form water flow and impacts is a prerequisite for developing a method to determine good river functioning. This is particularly relevant for watercourses subject to anthropogenic transformation [64]. Hydromorphological impacts affect rivers, riparian areas and floodplains in different ways. Examples include bank protection with embankments, dike construction, and a growing share of impervious surfaces in the catchment area, which increase the rapidity of river floods [65, 66]. Hydrotechnical structures can have a negative impact on river continuity, distorting the status of ichthyofauna. Weirs, barrages, and various types of water thresholds or culverts effectively restrict or close the migration corridor for fish and other species [67–69]. Artificial barriers and various dams also affect ecosystem functioning through changes in water temperature, oxygen concentration or nutrients and suspended organic matter [70]. The ecological status is negatively affected by maintenance works on the river. Weed cutting, removal of littoral vegetation, woody debris from the riverbed and banks, and dredging and modification of the riverbed affect macrophytes, ichthyofauna and macroinvertebrates [71]. The intensity of these treatments depends on the scope of the works undertaken, their scale, and the timing and technology used [72].

Once the significant factors have been selected for the hierarchical tree, the next step of the AHP method is to compare the factors in pairs with each other. A nine-point scale developed by Satty [73] is used for this purpose. The weights of the individual factor comparisons were used to construct the matrices. A characteristic feature of the prepared matrices is that the diagonal values equal 1. The values above the diagonal are filled in with the results of pairwise comparisons of individual factors. At the same time, the values under the diagonal are their inverses. The values of the local vectors were obtained from the matrix solutions. In order to calculate the solution values of the matrix in the first step, the geometric mean of each row of the matrix of pairwise comparisons was calculated from the relation.

ri=∏j=1n(aij)12 (2)

where,

a_ij_(_i,j_ = 1,… n are the row and column numbers of the matrix)

n—number of criteria, matrix dimension

r_i_—geometric mean of each row for the pairwise comparison matrix

In a second step [74], each criterion or solution’s weights (w_i_) were calculated.

$$w_{i}=\frac{r_{i}}{\sum_{j}r_{j}}$$ (3)

where,

w_i_—weights of each criterion

r_i_—geometric mean of each row for the pairwise comparison matrix

Each matrix was checked for correct calculation. The maximum eigenvalue (λ_max_), and the Consistency Index (CI), Random Index (RI), and Consistency Ratio (CR) were used for this. The maximum eigenvalue should be approximately equal to the dimension of the matrix. Random Index depends on the dimension of the matrix, and its values can be found in Jacob and Subramoniam [75]. The CR values were then calculated from the CI and RI values.

$$\lambda_{max}=\frac{\sum_{i=1}^{n}\lambda_{i}}{n}$$ (4)

where:

*n*–dimension of the matrix;

λ–eigenvalue of the matrix for the i-th row.

The Consistency Index (CI) is calculated according to the following equation:

$$CI=\frac{\lambda_{max}-n}{n-1}$$ (5)

where:

n–dimension of the matrix;

λ_max_−maximum eigenvalue.

The Table 3 summarizes the RI values depending on the eigenvalue of the matrix.

**Table 3:** Random index values depend on the matrix eigenvalue [76].

n	1	2	3	4	5	6	7	8	9	10	11	12
RI	0	0	0.52	0.89	1.11	1.25	1.35	1.40	1.45	1.49	1.52	1.54

Because of the partial lack of objectivity of the assessments, the Consistency Ratio *CR* is calculated:

$$CR=\frac{CI}{RI}$$ (6)

where:

CI–Consistency Index

CR–Consistency Ratio

RI–the value of the Random Index

If CR > = 0.1, this indicates inconsistent evaluation [77, 78]. When the CR value in the matrix is less than 1, the matrix solution can be said to be correct. The resulting local vector is the solution of the matrix, based on which the factors or solutions were prioritised. After checking the values obtained from the calculation of each matrix, further analysis was carried out, and the values of the global vectors were determined [79]. Global vectors were calculated by multiplying the local vector from a given level by the global vector from the level above. Based on the calculation scheme presented above, the hierarchy of individual solutions and their weights were determined. A summary of parameters maximum eigenvalue (max), Consistency Index (CI) and Consistency Ratio (CR) for Level II, III and IV matrix is presented in S2 and S3 Tables.

Multiple-Criteria Decision-Making (MCDM) methods are beneficial for analysing problems in various fields. Many MCDM methods can be found in the literature. However, they differ in various scales of comparison, methods of calculation and the type of results obtained. In the analyses conducted by the authors, the AHP method was chosen. Many authors from different fields have been using it for a long time. This is confirmed by the analyses conducted by Emrouznejad and Marra [80], containing publications in which the AHP method was applied. Based on the study conducted from 1979 to 2017, it was found that the method was implemented in 8441 articles. The highest increase in publications using the AHP method was observed between 2005 and 2017. According to Løken [81], Emrouznejadiand Marra [80], the AHP method used in this work is flexible and intuitive. It allows quantitative and qualitative criteria analysis, checks consistency between responses for a given matrix, and synthesises judgments to determine which variables have the highest priority. The basic idea behind this method is that it is more convenient to perform pairwise evaluations rather than to evaluate solutions directly [82]. Furthermore, Macharis et al. [83] found that a big advantage of the AHP method, as opposed to, e.g. the PROMETHEE method, is the possibility to give specific weights in a simple way. The PROMETHEE method, on the other hand, requires a great deal of experience from the person carrying out the analyses with this method.

The weights of individual elements determined with the AHP were used in the Comprehensive Assessment Method for Lowland Rivers (CALR). Subsequently, detailed rules for assessing with CALR were developed, and an assessment sheet using the weights.

## 3. Results

### 3.1. Results of fieldwork

#### 3.1.1. Macrophytes assessment

The results obtained based on the Macrophyte Method for River Assessment indicate the river’s good condition. The MRI index ranged from 36.9 to 42.5. The river is characterised by a high degree of naturalness, especially along its source section, up to Lake Worowskie. Riparian forests and wetlands are the dominant landscape there. Further, the catchment area changes its character to agricultural. However, this area is dominated by grassland cut by forests. Only right in front of the reservoir in Przebędowo is arable land. Behind the reservoir, the river flows through the urbanised area of Murowana Goślina. However, the pressure from the building side is insignificant because the Trojanka River flows largely through the park and the areas adjacent to the gardens and through wetlands. Only a small section of the river flows alongside residential buildings. Therefore, anthropogenic pressure in this place did not significantly affect the MRI index. Even though the point "behind the Przebędowo reservoir" index was the lowest among the respondents, it was still in the second class. The indicator slightly increases at the "Mściszewo" point, where the river changes from agricultural to forested use. The estuary section is natural.

During bioindication studies, 53 species of macrophytes were registered, including 3 species of algae, 1 species from the group of ferns, 1 moss and 48 species of vascular plants, of which 29 species are indicator species. On average, 18 plant species were recorded at the site—the most in the source section "Zielonka" (22 species) and the least in the estuary section in Mściszewo. The largest indicator species are recorded at the point "behind the Przebędowo Reservoir" (16 species). Although the river has a natural character to a large extent, hydrotechnical modifications could have caused a reduction in the quality of the habitat. In its upper section, Lake Zielonka is dammed up, which may modify the condition of the water habitat. The Przebędowo dam reservoir and the accompanying structure could modify the biodiversity of the Trojanka River. Undoubtedly, the urbanised area of Murowana Goślina also impacts biodiversity. The presence of such elements as culverts could also have a small impact on the ecological status of the river. The pressure from the factors mentioned above was not significant, however, because the condition of the waters indicates the second class.

#### 3.1.2. Hydromorphological assessment

Hydromorphological studies of the Trojanka River using the River Habitat Survey method show greater differentiation than for the MRI index. The HQA index classified the river as class I. However, the factor determining the hydromorphological state was the HMS index, which defined the rank of anthropopressure. The lowest anthropopressions were recorded at T1 (source), T3 in Gębice and T5 (estuary). The greatest anthropopressure occurred at the point “behind the Przebędowo Reservoir”, where the river flows into the urbanised area of Murowana Goślina (Table 4).

**Table 4:** Macrophyte and hydromorphological indicators of the analysed sections of the Trojanka River based on the MMRA and RHS methods.

River code	Name of points	MRI	Class of ecological status MRI	Values of habitat quality assessment (HQA)	Naturalness class (HQA)	Values of habitat modification score (HMS)	Modification class (HMS)	Hydromorpho-logical state class
T1	Zielonka	39.1	II	67	I	1	I	I
T2	Głęboczek	38.8	II	64	I	8	II	II
T3	Gębice	42.5	II	60	I	0	I	I
T4	Behind the Przebędowo Reservoir	36.9	II	70	I	9	III	III
T5	Mściszewo	38.0	II	66	I	0	I	I

#### 3.1.3. Water quality assessment

The water quality was assessed based on the data from monitoring of the Regional Environmental Protection Inspectorate (REPI) in Poznań from 2008–2020 in three cross-sections characterising the upper, middle, and lower course of the Trojanka watercourse. The analysed cross-sections were located in three types of use: natural area, urbanised area, and agricultural area. The water quality results in the upper part of the watercourse concerned forest areas of the Zielonka Forest (PLH300058 Uroczyska Puszczy Zielonka). This measurement point is marked as W1 (52°35’33.6"N 17°04’14.9"E) on the map in Fig 1. The other results were related to the point W2 “behind the Przebędowo reservoir” (52°35’07.4"N 17°00’55.4"E), which is located in the central part of the watercourse in the immediate vicinity of agricultural land, while the lower part of the water intake is represented by the control point in Raduszyn–W3 (52°34’33.3"N 16°59’21.6"E), which is the most anthropologically transformed part of Trojanka River. The conducted water quality tests confirmed the poor condition of the Trojanka watercourse. Microbiological indicators such as Coliform bacteria, Escherichia Coli and Enterococci classify the water to a bad state (V quality class) [26]. During the research, water quality deteriorating tendencies along the course of the watercourse were noticed. Regarding the content of nutrients (total nitrogen, phosphorus), the water in the Trojanka watercourse was classified as class II and III [27]. The lowest concentrations of nutrients were recorded in the upper section of the watercourse, which flows through the forest areas of the Zielonka Forest. The water quality is significantly deteriorating in the central part of the watercourse, right behind the Przebędowo reservoir. When assessing water quality in terms of the classification of the state of surface water bodies and environmental quality standards for priority substances [28], the water of the Trojanka watercourse should be classified as out-of-class water (bad condition). The most abnormal water quality parameters were recorded in the lower section of the watercourse. As a result of the research, the water quality of the Trojanka watercourse was assessed at 1.0.

#### 3.1.4. Hydrological assessment

The first element assessed in this section was meteorological conditions, including precipitation (PP). Based on data from the meteorological station at Zielonka Arboretum for the last 15 years, it was found that the precipitation for the Trojanka River was 560 mm. Thus, within the hydric significance indicator (HS), 2.0 points were awarded for this element. The land cover analysis conducted in QGIS showed that the Trojanka River area is dominated by forests (63.32%). Moreover, the river also flows through meadows (16.63%), arable fields (15.24%), and built-up areas (4.81%). Next, forest ecological status (F) was assessed based on the forest damage level map included in the Forest Status Report [84]. It was found that the Trojanka River area has medium damage (20.1–25%). Thus, according to the methodology, 1.0 point was awarded for this parameter. Soil conditions in the HS method were divided into soil types according to wetness index (Swet), and soil texture (Stext) was assessed based on the analysis of the soil map of the Trojanka River. Thus, the Stext parameter was assessed at 2.0 points. In Swet, the Trojanka catchment is dominated by brown earth soils (Cambisol). Following the methodology, the Swet parameter was assessed at 0.0 points. A geological formations map was used to assess another parameter—ground transmissivity (T). Based on its analysis, the transmissivity was assessed as medium (0.0 points). The last parameter in the hydrological assessment was lake retention (LR). In order to conduct it, bathymetric measurements were made of all lakes through which the Trojanka River flows. The results obtained are summarised below:

- Huckie Ponds, water volume 20 806 m^3^, score 0.5;
- Zielonka Lake, water volume 47 341 m^3^, score 0.5;
- Zielonowski Pond, water volume 6 648 m^3^, score 0.5;
- Głęboczek Lake, water volume 29 857 m^3^, score 0.5;
- Leśne Lake, water volume 250 841 m^3^, score 1.0;
- Głębocko Lake, water volume 51 395 m^3^, score 0.5;
- Worowskie Lake, water volume 386 702 m^3^, score 1.0;
- Przebędowo Reservoir, water volume 162 350 m^3^ [85], score 0.5;
- Raduszyn Reservoir, the micro reservoir currently exploited as a peat deposit, score 0.5.

Based on the measurements, three lakes (Leśne Lake, Worowskie Lake and Przybędowo Reservoir) can be classified as small reservoirs (1.0 points). The remaining lakes on the Trojanka River are micro reservoirs (0.5 points). The scores obtained from the individual parts of the hydrological assessment enabled the calculation of the HS indicator’s final value in the QGIS environment from the equation. The Trojanka River received 22.1 points which allowed it to be classified as excellent (5) in terms of hydrological assessment.

#### 3.1.5. Hydrotechnical structures assessment

During the fieldwork carried out on the Trojanka River, 30 water structures were identified. Culverts dominated among them. Moreover, the structures included monks, headgates, and sills. Their technical condition was assessed based on individual structure elements’ measurements. The results are summarised below:

- good technical condition (score 5): 13 structures (43.33% of all constructions on the Trojanka River);
- satisfactory technical condition (score 3): 8 structures (26.67% of all constructions on the Trojanka watercourse);
- unsatisfactory technical condition (score 1): 9 structures (30.00% of all constructions on the Trojanka River).

On the Trojanka River, 43.33% of water facilities are in good technical condition. However, a slight majority of structures—56.67%—are in poor technical condition (satisfactory and unsatisfactory). The parameters determining the negative assessment were most often the degree of siltation and the depth of cracks in abutments. The hydrotechnical constructions on the Trojanka River are mostly neglected and undergo gradual decapitalisation. The process of devastation is particularly noticeable on the abutments and pillars of the structures, where deep cracks in the concrete occur. On some structures, the damage is so extensive that it exposes structural elements (reinforcement) or is overgrown by vegetation. As a result of the conducted research and calculations, the technical condition of all hydrotechnical constructions on the Trojanka River was assessed at 3.0, which is equivalent to a satisfactory condition.

#### 3.1.6. Hydrodynamic assessment

The analysed Trojanka River was divided into three sections. The forest section from the spring to Lake Worowskie was mainly characterised by undeveloped curvature of arcs (grade 1), except for a few small river sections characterised by developed curvature. The agricultural section from Lake Worowskie to Przebędowo Reservoir was characterised by undeveloped curvature of arcs (grade 1), similarly to the urban section. Finally, based on the analysis of the entire Trojanka River, the curvature of the water arcs was undeveloped (grade 1).

The analysis of the river bed and the vegetation present in it indicates that the average roughness coefficient in the forest section of the river is 0.03 (grade 1). The agricultural section is characterised by an average coefficient of roughness of 0.035 (grade 2). Finally, in the urban section, it can be observed that the average roughness coefficient is 0.035 (grade 2). The entire Trojanka River analysis classified it in terms of channel and vegetation to a rating of 2.

The next step was to analyse and relate the flows to the environmental flows. First, the multi-year average flow was determined by 0.68 m^3^·s^-1^. According to Michalec and Zwolenik [34], flow magnitude analyses are performed for two periods, October—March and April—September. The measured flow value in November was 0.42 m^3^·s^-1^ (grade 3). The measured flow value in July was 0.38 m^3^·s^-1^ (rating 3). In terms of environmental flows for the whole year and the entire river length, a rating of 3 can be given. The average rating for the individual elements of the hydrodynamic assessment is 2.

### 3.2. Results of the AHP method

**Fig 6:**
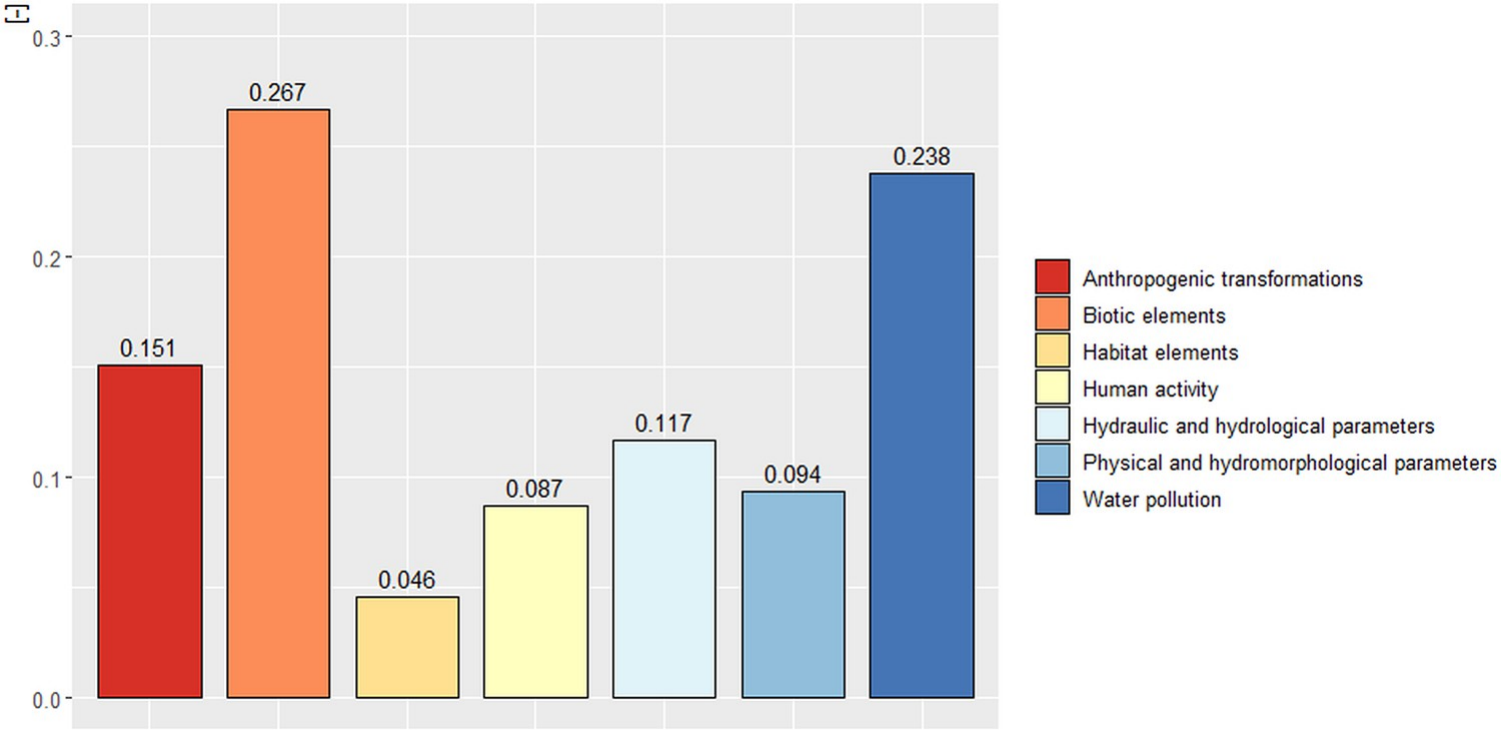
Global vector values for level II of the hierarchical tree.

As a result of the calculations carried out with the AHP method, the values of global and local vectors for particular factors of the hierarchical tree were obtained. The values of all calculated global vectors are presented in the tree diagram provided in the S1 Fig. Furthermore, Fig 6 shows the results of the matrix solution for level II of the hierarchical tree.

**Fig 7:**
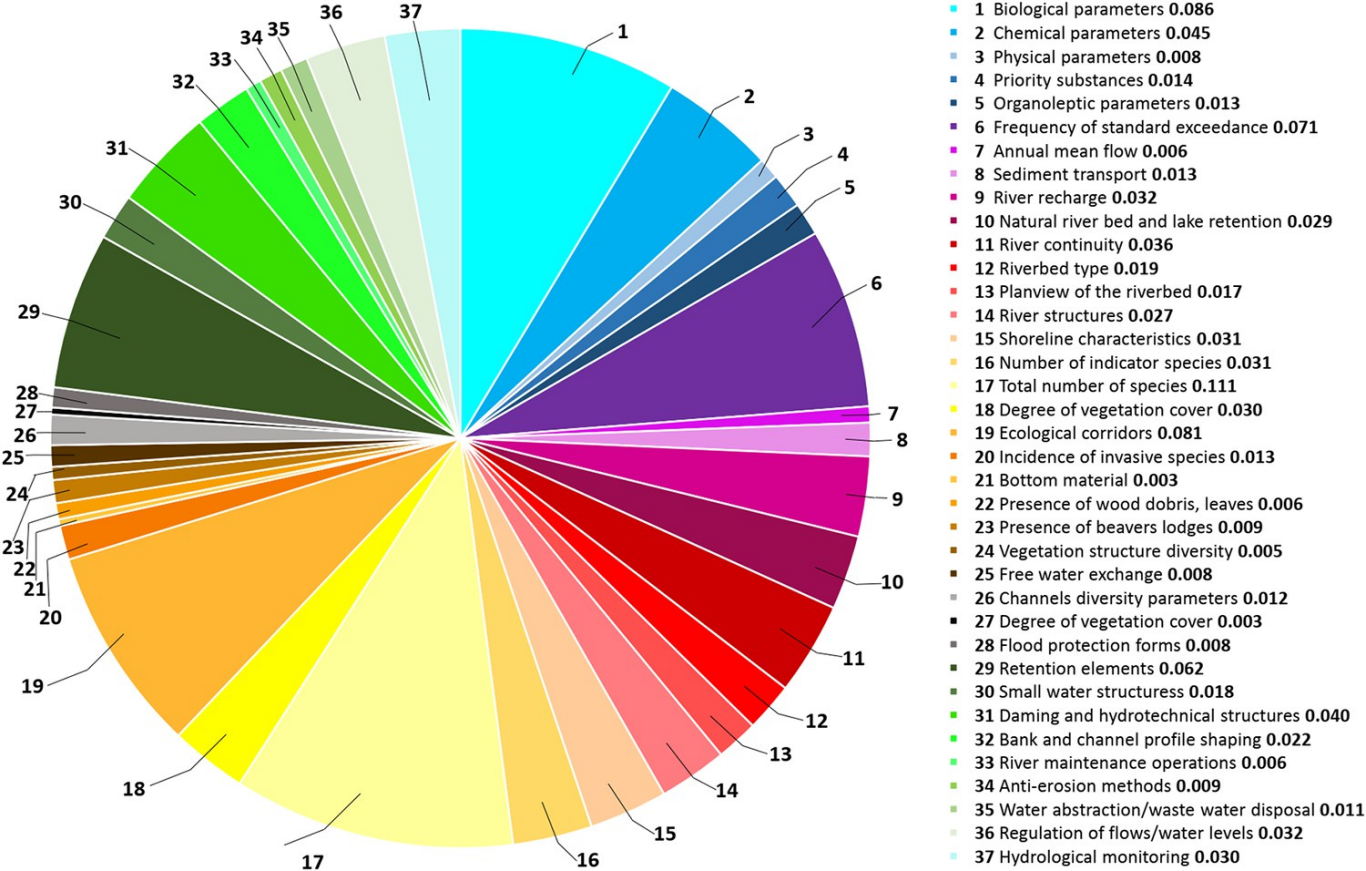
Global vector values of level III of the hierarchical tree.

At level II of the hierarchical tree, biotic elements were the most crucial factor, with a value of (0.267). They were followed by water pollution (0.238). The factor of least importance in the analysis was habitat elements (0.046). Next, the values of local vectors at level III were determined for individual branches of the tree. Finally, the obtained values were converted to the global vector (Fig 7).

The highest value of the global vector for Level III was obtained for the total factor number of species (0.111), which originated from biotic elements. The second most important parameter in terms of global vector value was the biological parameters (0.086), an essential element in water quality assessment. The third most important factor was ecological corridors, which play a significant role in the migration of various species and maintaining the continuity of biological life in the river. Finally, the lowest values were given to factors related to habitat elements: degree of vegetation cover (0.003), bottom material (0.003), and diverse vegetation structure (0.005).

**Fig 8:**
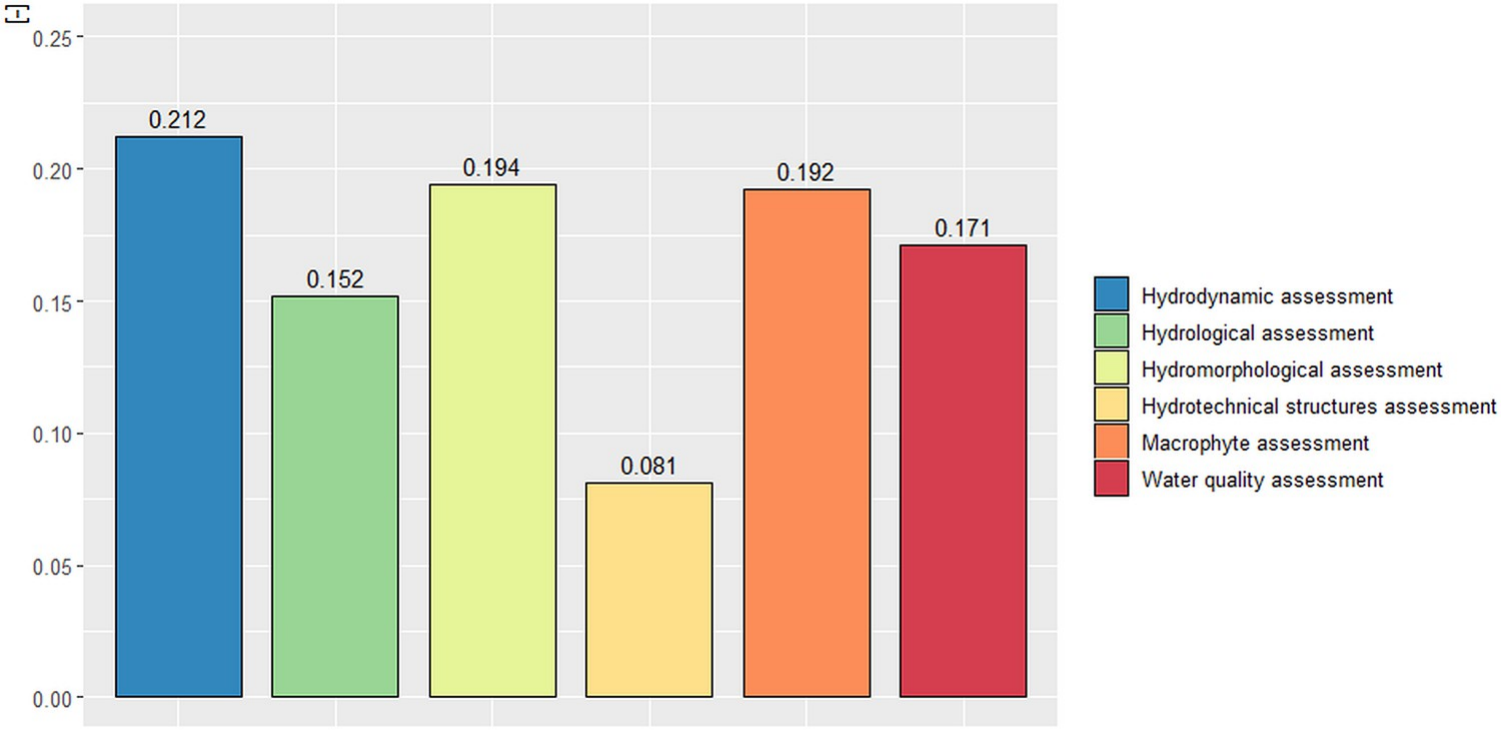
Global vector values for level IV of the hierarchical tree.

The most important results were the global vector from level IV of the hierarchical tree (Fig 8). This level included six different evaluation elements used in the Comprehensive Assessment of Lowland Rivers.

The highest vector value was obtained for hydrodynamic assessment (0.212), which thus became the most crucial element in the Comprehensive Assessment of Lowland Rivers. The following two elements with similar importance were the hydromorphological assessment (0.194) and the macrophyte assessment (0.192). The water quality assessment received a value of 0.171, and the hydrological assessment obtained a value of 0.152. The lowest global vector value at level IV of the hierarchical tree was found for the hydrotechnical structure’s assessment (0.081). The vector values calculated above are the essential part of the results from the AHP in this publication. They made it possible to classify and assign weights to individual elements included in the Comprehensive Assessment of Lowland Rivers (CALR). Therefore, the values obtained result from expert analysis and mathematical calculations and are appropriate for implementation in a Comprehensive Assessment of Lowland Rivers method. Furthermore, they successfully solve the complex problem of classifying individual evaluation elements.

### 3.3. Comprehensive Assessment of Lowland Rivers

The main reason for developing a new river assessment method was the need to combine several aspects and factors to correctly reflect the river’s condition. Current methods tend to focus on one aspect of river function (e.g. physicochemical status of the water) and do not attempt to integrate and assess all factors affecting the river. In developing the Comprehensive Assessment of Lowland Rivers (CALR) methodology, existing proven methods for assessing individual parameters were used. The AHP method was used to select suitable parameters for CALR and above all, to classify their importance. As a result of analyses and calculations using this method, weighted values were obtained for the six individual assessment elements (water quality, hydrology, hydrodynamics, hydromorphology, macrophytes, and hydrotechnical structures assessment), which were implemented in the Comprehensive Assessment of Lowland Rivers methodology. Based on that, a form for carrying out CALR was designed (Table 5). The proposed method and the created form can be used for lowland streams with a catchment area of 10–100 km^2^ and small lowland rivers with a catchment area of 100–1000 km^2^ in the lowland landscape type <200 m a.s.l, regardless of the type of substrate (organic, loess, loamy, sandy, gravel) with a high degree of naturalness, or under the influence of anthropogenic pressure (agriculture, buildings, water or forest management). The created form can be successfully applied to any lowland river. The authors have tried to construct it to be relatively easy to fill in and allow a single result to be obtained. Using the created form in this study, an assessment was carried out for the example of the Trojanka River. The results obtained from individual methods were used and are summarised in Table 5.

**Table 5:** Results of individual parts of the Comprehensive Assessment of Lowland Rivers obtained for the Trojanka River.

Element of the Comprehensive Assessment of Lowland Rivers (CALR)	Obtained Score
Hydrodynamic assessment	2.0
Hydrological assessment	5.0
Hydromorphology assessment	3.0
Hydrotechnical structures assessment	3.0
Macrophytes assessment	4.0
Water quality assessment	1.0

The results show that the hydrology parameters were rated the highest on the Trojanka River (score 5.0). On the other hand, the lowest-scored element was water quality. Therefore, it can be concluded that this river has a huge water retention potential, but immediate actions should be taken to improve the water quality. In addition, for the better functioning of the watercourse, the existing hydrotechnical structures, the condition of which was assessed at 3.0, must be renovated and maintained. Proper maintenance of the structure affects the hydrodynamics of the watercourse, which was rated low (2.0), and also increases the water retention capacity. Bioindication studies performed using the MMRA method indicate a good condition of the Trojanka River in all examined points, which is mainly influenced by natural areas within the river catchment. The slight decrease in the ecological status is mainly due to urbanized areas. Biological parameters are one of the most important parameters; they were rated 4.0 in the CALR method. Hydromorphological tests performed with the RHS method indicate a slightly larger qualitative differentiation. The examined points were assessed between the I and III class of hydromorphological state. The method reacted to a large hydromorphological transformation at the point “behind the Przebędowo Reservoir”, at the level of the urbanized city of MurowanaGoślina. The hydromorphological elements were rated 3.0.

The obtained results for each element of the Comprehensive Assessment of Lowland Rivers were transferred to the assessment form (Table 6). Then, to obtain the final assessment by the CALR method, the results of each element were multiplied by the weights obtained using the AHP method. The classification of the Trojanka River was based on the 5-point scale adopted by the CALR method (Table 7).

**Table 6:** Assessment form for carrying out the Comprehensive Assessment of Lowland Rivers (CALR).

**BASIC INFORMATION ABOUT THE ANALYSED RIVER**		
River name	Trojanka	
River length [km]	20.8	
Catchment area [km^2^]	145.34	
**COMPREHENSIVE ASSESSMENT OF LOWLAND RIVERS (CALR)**		
**Element**	**Element weight**	**Obtained score**
Hydrodynamic assessment	0.212	2
Hydromorphology assessment	0.194	3
Macrophyte assessment	0.192	4
Water quality assessment	0.171	1
Hydrological assessment	0.152	5
Hydrotechnical structures assessment	0.081	3
**RESULTS**		
Final evaluation by CALR		**2.948**
The river condition was classified as		*moderate*

**Table 7:** CALR river rating scale.

Point range	River condition
0.0–0.99	bad
1.00–1.99	poor
2.00–2.99	moderate
3.00–3.99	good
4.00–5.00	very good

The Trojanka River received 2.948 points which classifies it as a river in moderate condition according to the Comprehensive Assessment of Lowland Rivers (CALR). A blank assessment form is included in the supplementary materials to support the evaluation of other lowland rivers using the CALR method (S4 Table).

## 4. Discussion

Rivers are a complex ecosystem, sensitive to the pressure of many factors and dependent on the use of the catchment area [86]. Therefore their assessment requires considering many parameters, both natural, and hydraulic, and including anthropopressure. However, the river assessment methods currently used in science often focus on only one aspect, such as the physico-chemical quality of water or the hydromorphological assessment. Such inventory and valorization lead to a narrow assessment covering only a part of a complex river ecosystem. Therefore, methods should be sought to enable an integral, objective, comprehensive assessment of the river. The system presented in this paper gives a very comprehensive view of the river ecosystem’s values, as it considers several elements characterising the biotic and abiotic elements of the river habitat. So far, despite the known problems with the objective assessment of rivers, no research methodology has been proposed in such a dimension and treating the river system in such a broad way. This method considers habitat, hydrodynamic and hydrological elements, and anthropressure to watercourses and includes human activity in the catchment area. River monitoring with a comprehensive methodology aims to provide knowledge about the ecological status (or ecological potential) necessary to manage water in the river basins and meet the environmental objectives. The method enables planning of protection against eutrophication and anthropogenic pollution, assesses the habitat’s ability to exchange biological material, and also characterises the dynamics of this habitat.

One of the most crucial water management tasks is restoring aquatic ecosystems to a near-natural state by protecting against water pollution. Therefore, it is not surprising that most scientific studies regarding rivers focus on water quality assessment [87]. In this aspect, the classification of surface water status is based on analysing the results of measurements of chemical pollutants, including the so-called priority substances. The assessment consists of comparing the obtained results with environmental quality standards. Water has a good chemical status when no calculated concentration values exceed the standards. In the classical assessment method, the water’s purity was analysed, i.e. primarily the use aspect (e.g. drinking water). The water quality assessment aimed to provide knowledge on the condition of the water and the measures taken to improve its condition and protect it from pollution. The actions carried out focused on protecting the river against eutrophication, which is a consequence of pollution from household and agricultural sources. Moreover, they also focused on protection against industrial pollution associated with salinity and the content of substances particularly harmful to the aquatic environment. Following the Water Framework Directive guidelines, this assessment should be based on biological studies supplemented by chemical analyses as background for the final assessment of the ecological status of the river. Furthermore, in the global literature, the most commonly used indices to assess water quality are the Water Quality Index (WQI) and the National Sanitation Foundation’s Water Quality Index (NSFWQI) [88–92]. The evaluation based on the abovementioned indices consists of experts’ selection or legislation of relevant parameters and weights to each parameter. For the NSFWQI, nine parameters are used: pH, temperature, turbidity, dissolved oxygen, fecal coliform, biochemical oxygen demand, total phosphates, nitrates, and total solids. The final index values are then calculated using specific formulas. The score obtained from the calculation (0–100) is compared to the five-point NSFWQI rating scale excellent (91–100), good (71–90), moderate (51–70), low (26–50) and bad (0–25), [93, 94]. Another index commonly used in river assessment is the Water Pollution Index (WPI). It is also used to classify water quality based on calculated threshold values [95, 96]. Water quality is also considered in the comprehensive river assessment method developed by the authors of this manuscript. Based on the analysis of the results of the multi-criteria decision support (AHP) method, the weight of this aspect was found to be 0.171, which is the fourth most crucial evaluation factor.

Furthermore, scientists have also noted the need to develop a new Fish-Water Pollution Index (FWPI). It was created based on the provisions of the Water Framework Directive. Its main objective is to assess parameters that ensure the water quality necessary for fish existence [97]. The Water Framework Directive 2000/60/EC (WFD) [2] obliges all European Union countries to rationally use and protect water resources following the principle of sustainable development and achieving good waters’ ecological status. It is determined based on biological, physico-chemical, and hydromorphological parameters. In order to assess the ecological condition of the watercourses, it is also necessary to prepare an inventory and hydromorphological valuation. Over the years, many different methods of hydromorphological valorization of rivers have been developed. The Länderarbeitsgemeinschaft Wasser (LAWA-vor-Ort) is considered the most refined [98]—German system and Systemed’Evaluation de la Qualité du Milieu Physique (SEQ-MP)—French system [99]. Particular systems differ in the number and type of registered morphological elements and in the field research procedure. Among the most popular methods used in Poland the following can be mentioned River Habitat Survey (RHS)—the British method developed, and Hydromorphological Monitoring of Rivers (MHR)—the Polish method [100]. In addition to the two mentioned, other ones have also used assessment of the quantitative and morphological status of surface water bodies in order to determine strongly changed water bodies—the Polish method [101], and a comprehensive (geoecological) method of assessing the natural values of a large river valley—Polish method [102]. Concerning the hydromorphological assessments of rivers, an analysis was carried out for 1983–2013, which showed that there are more than 121 different methods. Despite such a wide scope, it has been found that many of them do not take into account several important physical factors. Moreover, the lack of an interdisciplinary approach to river condition assessment to integrate many different aspects was noticed [103]. It also noted that different indicators and data are considered in different EU Member States for the hydromorphological assessment of rivers. In practice, experts in various aspects of the aquatic environment use their own modified research methodologies, relying on their own experience and adjusting the existing recommendations to the specificity of watercourses and their catchment areas. This is due to, inter alia, the difficulty of finding common criteria for assessing rivers of extremely different sizes or characteristics. The RHS method focuses on the riverbed and the riverbed zone of the watercourse, while the processes operating here usually reflect the state of the environment in the entire catchment area, i.e. both the valley system and the slope system. Therefore, the analysis of individual sections of the watercourses must be supported by the analyzes of the state of the environment in the partial catchment located above the section due to the large diversity of the catchment’s lithological conditions and the level of anthropopressure. The processes of erosion, transport and deposition of river sediment on flysch have different dynamics and similar processes on the crystalline bedrock. Human interference in the system of small watercourses in various parts of Europe and the world is also different. One of the most frequently mentioned disadvantages of the RHS system is that it does not consider such elements as the hydrological regime. For these reasons, the obtained RHS results without additional interpretation may be controversial [104]. Scientists emphasize the need to standardize the methodology [105].

Moreover, there are many methods of determining the degree of river degradation. Most of them relate to the eutrophication process (total phosphorus concentration). They use plant bioindicators for this purpose. Based on the identified species of macrophytes and the degree of their coverage, indexes are calculated to assess the ecological state. Among the best-known and most used methods can be mentioned six macrophyte metrics: Mean Trophic Rank (MTR) [106] and RMNI—River Macrophyte Nutrient Index [107]–British methods, Trophäe-Index Macrophyten (TIM) [108], Reference Index (RI) [109]–German methods, Indice Biologique Macrophytiqueen Rivière (IBMR) [110]–French method. The research on the ecological status of rivers in Poland is most often carried out based on the Polish method (Macrophyte Method for River Assessment), adapted to the habitat conditions of this climate zone. This method is the official Polish method of macrophyte monitoring [111], in line with the recommendations of the European Committee for Standardization [112]. Despite its many advantages, the macrophyte method has several disadvantages. It does not account for the periodic deterioration of the water condition caused, e.g. by spontaneous discharges of pollutants or surface runoff from farmland. Macrophytes react with a long delay to changes in the aquatic environment, indicating a permanent state. Additionally, as observed by the authors of this study, in the case of highly degraded waters, where the standards for biogenic parameters are exceeded even several hundred times, the water status based on macrophytes was described as weak or even moderate, but not bad, and such should be demonstrated. In the comprehensive method of lowland river assessment proposed by the authors of this study, the river assessment using macrophytes, apart from the hydrodynamic (0.212) and hydromorphological (0.194) assessment, has a high rank (0.192), which is in line with the recommendations and guidelines of the EU Water Framework Directive. However, it is of particular importance in combination with other parameters that concern not only the chemical state of the river and biological potential but also a number of other characteristics, such as river continuity, water retention, or the presence and condition of hydrotechnical structures.

Environmental flow is also very often assessed in river studies. Under this heading is defined as the water needed within managed rivers to support critical ecological processes and the human well-being provided by the ecosystem [113]. It has been noted that the central pillar of sustainable water management should be the conservation and protection of environmental flows and adequate water quality [114]. Preserving environmental flows allows for mitigating the effects of river regulation and thus allowing fish migration [115]. Furthermore, it has been proven to link preserving environmental flow and fish spawning [116]. The assessment of environmental flows is critical when water resources are used for industrial and economic purposes and when planning the construction of hydrotechnical structures such as weirs or hydroelectric power plants. It should be borne in mind that any water abstraction for energy purposes should occur at appropriate flows necessary for environmental protection. Scientific studies have noted significant differences in the obtained results of environmental flows obtained by different calculation methods [117]. Furthermore, the analyses showed that there are 207 different methods for describing environmental flow in the world. These methods are based on hydrology, hydraulic rating, habitat simulation, and holistic or combined methodologies [118]. A factor that directly affects environmental flow is damming structures and hydraulic structures that restrict water flow in a river. Therefore, researchers increasingly recognise the need for environmental assessment of currently functioning structures. An example of this is the classification of currently existing dams in China regarding their impact on environmental flow and the need to optimise their operation and regulation [119].

Another element assessed in the context of rivers is the geomorphological processes that determine the physical structure of rivers. River habitats’ spatial and temporal variability is related through channel morphology and substrate characteristics with the flow [120]. Flow hydraulics are strongly influenced by channel cross-sectional shape, channel roughness, and slope. In turn, channel morphology and substrate characteristics are influenced by debris transport processes, including transport dynamics and sediment characteristics. Therefore, many parameters related to bedload transport, suspended load, total transport, bank erosion depth, and water level are included in the Overall Weighted Indicator (OWI). Finally, OWI is calculated using the equation to calibrate numerical models. Channel meandering is a natural process that shapes the horizontal system [121]. This process is caused by the interaction of gravity, centrifugal, and frictional forces on each water particle. These cause the water level on the outer bank side to rise and the water on the inner bank side to fall. The resulting pressure difference causes water particles to move to the outer bank [56]. Field studies carried out on the Spree bend confirm the assumptions presented. The authors of the paper divide the cross-section into Pool (part of the cross-section on the outer bank side) and Riffle (part of the bank on the inner side) [122, 123]. The characteristics of water movement on the bend result in the deposition of sediment in the part of the cross-section on the inner bank side and an increase in water depth due to bottom erosion on the outer bank side [124]. Hydrodynamic assessment methods often require knowledge of complex software to calculate hydraulic parameters. In addition, it is necessary to collect a very large amount of initial and boundary data necessary to run the model [125, 126]. Any model of this type requires calibration and validation [127]. Many analyses indicate that river meandering supports biodiversity. The complex relationship between bed morphology and sediments transport favours a diverse mosaic of geomorphic units [128]. The work presented here uses a simple assessment methodology and can be used by anyone with adequate training. The hydrodynamic element in the CALR method received a weight of 0.212—the highest of all six elements analysed.

Furthermore, an important aspect when assessing hydraulic habitat is the influence of different water depths in the river channel on bedforms. Blois et al. [129] found that bottom flows are very important in shaping the bed. The exchange of mass and momentum near the bottom shapes openwork textures in gravels and the absence of ripples in coarse sands. Cluster analysis can be used for this type of analysis [130]. The HMID method can classify the hydromorphological heterogeneity of a river. This method makes it possible to classify a river section according to its degree of transformation [131, 132]. The environmental aspect is also linked to many other factors influencing the river. Recently, research has identified the need for a new index for rivers—the Happy River Index (HRI). This measure analyses the state of the river in the context of two aspects: river health and human well-being [133]. The integration of the anthropopressure aspect in river assessments is critical. Humans are constantly interacting with the environment and changing its state. Researchers see the construction of weirs as one of the reasons for the progressive deterioration of the ecological status of rivers [134]. It is well known that hydrotechnical structures directly affect water flow disturbance, alter habitats and hydrological and hydromorphological parameters of the river and impede fish migration. Leaving hydrotechnical structures in poor technical conditions can lead to the destruction of the structure and an ecological disaster. It should be borne in mind that the failure of a dam can cause flooding of adjacent areas and the consequent flow of pollutants into the river, destruction of habitats, and death of humans and many animal species. Therefore, it can be concluded that hydrotechnical structures should be considered in the overall assessment of a river due to their broad impact on the watercourse. However, none of the current river assessment methods considers this parameter. Some assessments only consider the technical condition of the hydraulic structure and its impact on the hydrodynamics of the watercourse while not assessing its environmental impact at the same time. In this method, it was found that the technical condition of the structure is a factor that must be taken into account but which is not the most crucial aspect of the river assessment. Its importance was estimated as 0.081, the last factor in importance.

The Comprehensive Assessment of Lowland Rivers method proposed by the authors considers the technical condition of hydraulic structures and hydrodynamic, hydrological, hydromorphological, environmental and water quality parameters. This allows us to assess a river as an integral whole where all aspects depend on and influence each other.

## 5. Conclusions

The assessment of a river should consider many parameters to reflect the actual condition of the watercourse. Despite many different methods, finding a holistic solution to perform this type of analysis in the literature is not easy. This paper has developed an innovative methodology for the Comprehensive Assessment of a Lowland River. It took into account an extensive range of parameters. The methodology used parameters such as the condition of hydrotechnical structures, water quality, hydromorphology of the river, hydrological and hydrodynamic parameters, and macrophyte evaluation. The multi-criteria decision support AHP method was used to prioritise the individual elements. As a result of the calculations performed, the highest value of the global vector and thus the highest value of importance in the CALR method was obtained for hydrodynamic assessment (0.212), followed by hydromorphological assessment (0.194), macrophyte assessment (0.192), water quality assessment (0.171), hydrological assessment (0.152) and the lowest value for hydrotechnical structures (0.081). Applying AHP in CALR guarantees the correctness of the ranking of factors in terms of importance due to the complex mathematical calculations performed.

The CALR method developed in this paper is the first river assessment method to combine a wide range of parameters covering both natural characteristics of the watercourse and transformations resulting from human impact. In this article, the authors included a detailed description of carrying out particular stages of CALR assessment, making it possible to perform it on any lowland river and implement it widely. It should be noted that CALR is a universal method and can be used to assess rivers worldwide. The authors point out that the presented methods of assessing the six elements (hydrodynamics, hydrology, hydromorphology, water quality, macrophytes, hydrotechnical structures) in CALR are only a suggestion and can be replaced by other corresponding methods. For example, in this study, water quality assessment was based on Polish standards, whereas in the CALR method, other indices such as the Water Quality Index (WQI) or Water Pollution Index (WPI) can also be used for quality assessment. The only thing to remember is that each CALR element must be classified according to a five-point scale, where 5 means the best assessment and 1 is the worst condition. This will allow the results to be correctly implemented into CALR. Another advantage is that assessing lowland rivers using the method proposed by the authors is not time-consuming and, despite analysing many factors, is relatively simple. In summary, the CALR classification of all lowland watercourses in the world will facilitate the process of inventory and enable comprehensive comparison between them.

## Supporting information

S1 Fig Map of the global vector values obtained using the AHP method. (TIF)

S1 Table Scales and grades used in the individual parts of the Comprehensive Assessment of Lowland Rivers. (DOCX)

S2 Table Summary of parameters maximum eigenvalue (F06C_max_), Consistency Index (CI) and Consistency Ratio (CR) for Level II and III matrix. (DOCX)

S3 Table Summary of parameters maximum eigenvalue (F06C_max_), Consistency Index (CI) and Consistency Ratio (CR) for Level IV matrix. (DOCX)

S4 Table Form for the Comprehensive Assessment of Lowland Rivers (CALR). (DOCX)
