# Supplementary figures and images for: The concept for innovative Comprehensive Assessment of Lowland Rivers
Source: PLoS One. 2023 Mar 9;18(3):e0282720. doi: 10.1371/journal.pone.0282720 (PMC9997953; doi:10.1371/journal.pone.0282720)

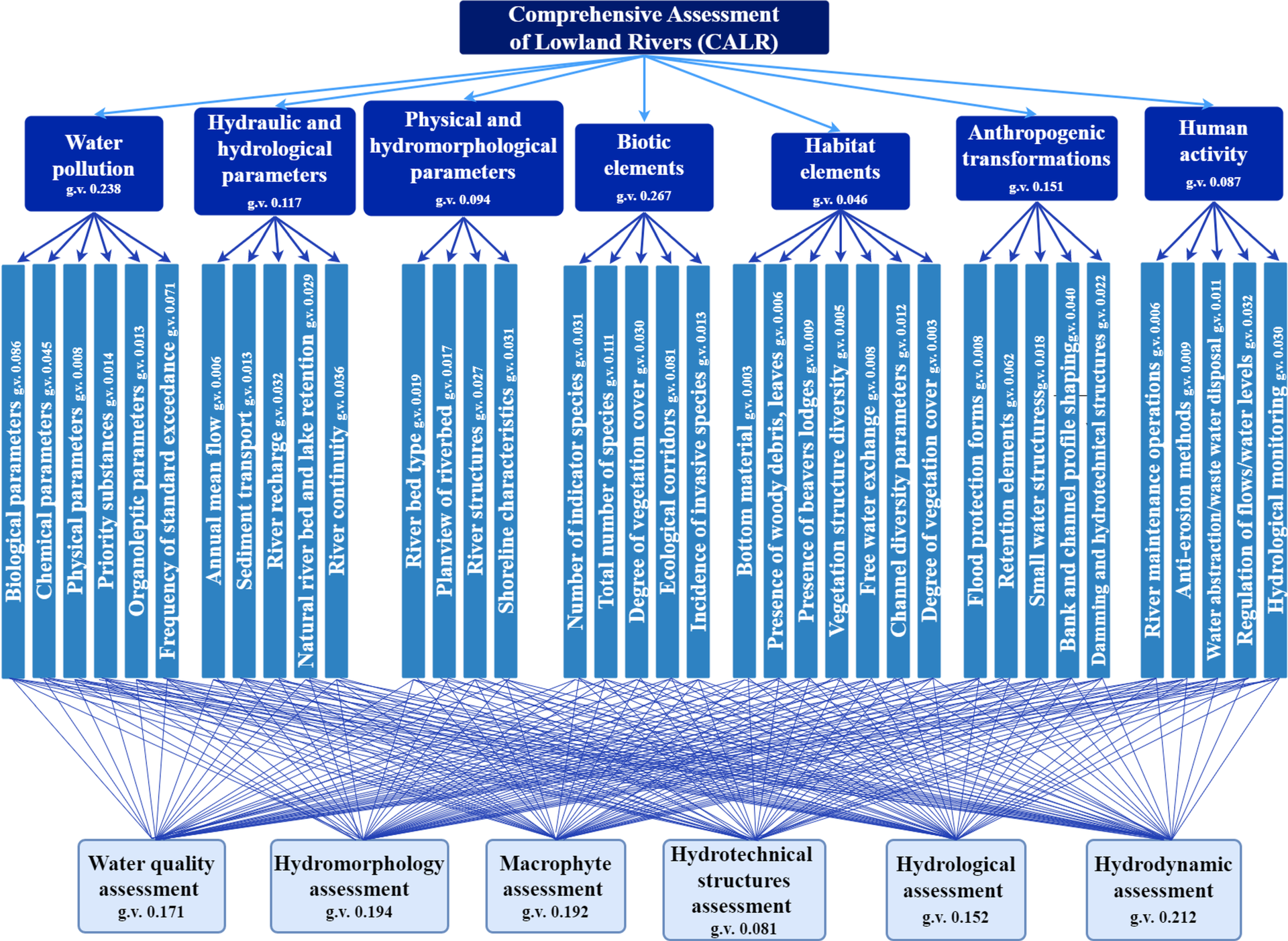

Supplement: S1 Fig — (TIF) [file pone.0282720.s001.tif]
