# Supplementary material for: The concept for innovative Comprehensive Assessment of Lowland Rivers
Source: PLoS One. 2023 Mar 9;18(3):e0282720. doi: 10.1371/journal.pone.0282720 (PMC9997953; doi:10.1371/journal.pone.0282720)
Supplement: S1 Table — (DOCX) [file pone.0282720.s002.docx]

**S1 Table.** Scales and grades used in the individual parts of the Comprehensive Assessment of Lowland Rivers.

| **Grading scale** |  | **CARL's part** | | | | | |
| --- | --- | --- | --- | --- | --- | --- | --- |
|  | **Rank** | **Macrophytes assessment** | **Hydromorphological assessment** | **Water quality assessment** | **Hydrological assessment** | **Hydrotechnical structures assessment** | **Hydrodynamic assessment** |
| **the highest rating** | 5.0 | very good status | I | I | excellent hydric significance | good technical condition | very good hydrodynamic parameters |
|  | 4.0 | good status | II | II | very good hydric significance | - | good hydrodynamic parameters |
|  | 3.0 | moderate status | III | III | good hydric significance | satisfactory technical condition | sufficient hydrodynamic parameters |
|  | 2.0 | poor status | IV | IV | average hydric significance | - | insufficient hydrodynamic parameters |
| **the lowest rating** | 1.0 | bad status | V | V | limited hydric significance | unsatisfactory technical condition | bad hydrodynamic parameters |
