# Supplementary material for: The concept for innovative Comprehensive Assessment of Lowland Rivers
Source: PLoS One. 2023 Mar 9;18(3):e0282720. doi: 10.1371/journal.pone.0282720 (PMC9997953; doi:10.1371/journal.pone.0282720)
Supplement: S2 Table — (DOCX) [file pone.0282720.s003.docx]

**S2 Table.** Summary of parameters maximum eigenvalue (λ_max_), Consistency Index (CI) and Consistency Ratio (CR) for Level II and III matrix.

|  | **λ_max_** | **CI** | **CR** |
| --- | --- | --- | --- |
| **Level II** | | | |
|  | 7.55 | 0.09 | 0.07 |
| **Level III** | | | |
| Water pollution | 6.21 | 0.04 | 0.03 |
| Hydraulic and hydrological parameters | 5.10 | 0.02 | 0.02 |
| Physical and hydromorphological parameters | 4.18 | 0.06 | 0.07 |
| Biotic elements | 5.35 | 0.09 | 0.08 |
| Habitat elements | 7.30 | 0.05 | 0.04 |
| Anthropogenic transformations | 5.13 | 0.03 | 0.03 |
| Human activities | 5.12 | 0.03 | 0.03 |
