# Supplementary material for: The concept for innovative Comprehensive Assessment of Lowland Rivers
Source: PLoS One. 2023 Mar 9;18(3):e0282720. doi: 10.1371/journal.pone.0282720 (PMC9997953; doi:10.1371/journal.pone.0282720)
Supplement: S3 Table — (DOCX) [file pone.0282720.s004.docx]

**S3 Table.** Summary of parameters maximum eigenvalue (λ_max_), Consistency Index (CI) and Consistency Ratio (CR) for Level IV matrix**.**

|  | **λ_max_** | **CI** | **CR** |
| --- | --- | --- | --- |
| **Level IV** | | | |
| Biological parameters | 6.12 | 0.02 | 0.02 |
| Chemical parameters | 6.10 | 0.02 | 0.02 |
| Physical parameters | 6.15 | 0.03 | 0.02 |
| Priority substances | 6.14 | 0.03 | 0.02 |
| Organoleptic parameters | 6.14 | 0.03 | 0.02 |
| Frequency of standard exceedance | 6.07 | 0.01 | 0.01 |
| Annual mean flow | 6.14 | 0.03 | 0.02 |
| Sediment transport | 6.13 | 0.03 | 0.02 |
| River recharge | 6.14 | 0.03 | 0.02 |
| Natural river bed and lake retention | 6.14 | 0.03 | 0.02 |
| River continuity | 6.13 | 0.03 | 0.02 |
| Riverbed type | 6.12 | 0.02 | 0.02 |
| Planview of the riverbed | 6.10 | 0.02 | 0.02 |
| River structures | 6.13 | 0.03 | 0.02 |
| Shoreline characteristics | 6.14 | 0.03 | 0.02 |
| Number of indicator species | 6.07 | 0.01 | 0.01 |
| Total number of species | 6.12 | 0.02 | 0.02 |
| Degree of vegetation cover | 6.12 | 0.02 | 0.02 |
| Ecological corridors | 6.01 | 0.00 | 0.00 |
| Incidence of invasive species | 6.00 | 0.00 | 0.00 |
| Bottom material | 6.10 | 0.02 | 0.02 |
| Presence of wood debris. leaves | 6.10 | 0.02 | 0.02 |
| Presence of beavers lodges | 6.13 | 0.03 | 0.02 |
| Vegetation structure diversity | 6.11 | 0.02 | 0.02 |
| Free water exchange | 6.03 | 0.01 | 0.01 |
| Channels diversity parameters | 6.13 | 0.03 | 0.02 |
| Degree of vegetation cover | 6.05 | 0.01 | 0.01 |
| Flood protection forms | 6.13 | 0.03 | 0.02 |
| Retention elements | 6.10 | 0.02 | 0.02 |
| Small water structuress | 6.13 | 0.03 | 0.02 |
| Daming and hydrotechnical structures | 6.14 | 0.03 | 0.02 |
| Bank and channel profile shaping | 6.12 | 0.02 | 0.02 |
| River Maintenance operations | 6.08 | 0.02 | 0.01 |
| Anti-erosion methods | 6.12 | 0.02 | 0.02 |
| Water abstraction/waste water disposal | 6.14 | 0.03 | 0.02 |
| Regulation of flows/water levels | 6.11 | 0.02 | 0.02 |
| Hydrological monitoring | 6.13 | 0.03 | 0.02 |
