# Supplementary material for: The concept for innovative Comprehensive Assessment of Lowland Rivers
Source: PLoS One. 2023 Mar 9;18(3):e0282720. doi: 10.1371/journal.pone.0282720 (PMC9997953; doi:10.1371/journal.pone.0282720)
Supplement: S4 Table — (DOCX) [file pone.0282720.s005.docx]

**S4 Table.** Form for the Comprehensive Assessment of Lowland Rivers (CALR).

| **BASIC INFORMATION ABOUT THE ANALYSED RIVER** | | |
| --- | --- | --- |
| River name |  | |
| River length [km] |  | |
| Catchment area [km^2^] |  | |
| **COMPREHENSIVE ASSESSMENT OF LOWLAND RIVERS (CALR)** | | |
| **Element** | **Element weight** | **Obtained score** |
| Hydrodynamic assessment | 0.212 |  |
| Hydromorphology assessment | 0.194 |  |
| Macrophyte assessment | 0.192 |  |
| Water quality assessment | 0.171 |  |
| Hydrological assessment | 0.152 |  |
| Hydrotechnical structures assessment | 0.081 |  |
| **RESULTS** | | |
| Final evaluation by CALR | |  |
| The river condition was classified as | |  |
